# GLUT1-mediated glycolysis supports GnRH-induced secretion of luteinizing hormone from female gonadotropes

**DOI:** 10.1038/s41598-020-69913-z

**Published:** 2020-08-03

**Authors:** Dequina A. Nicholas, Vashti S. Knight, Karen J. Tonsfeldt, Tomohiro Terasaka, Olivia Molinar-Inglis, Shannon B. Z. Stephens, JoAnn Trejo, Alexander S. Kauffman, Pamela L. Mellon, Mark A. Lawson

**Affiliations:** 1 Department of Obstetrics, Gynecology and Reproductive Sciences, and Center for Reproductive Science and Medicine, University of California, San Diego, La Jolla, CA USA; 2 Department of Pharmacology, University of California, San Diego, La Jolla, CA USA

**Keywords:** Metabolism, Homeostasis, Neuroendocrinology, Reproductive biology, Infertility, Endocrine reproductive disorders, Gonadal disorders

## Abstract

The mechanisms mediating suppression of reproduction in response to decreased nutrient availability remain undefined, with studies suggesting regulation occurs within the hypothalamus, pituitary, or gonads. By manipulating glucose utilization and GLUT1 expression in a pituitary gonadotrope cell model and in primary gonadotropes, we show GLUT1-dependent stimulation of glycolysis, but not mitochondrial respiration, by the reproductive neuropeptide GnRH. GnRH stimulation increases gonadotrope GLUT1 expression and translocation to the extracellular membrane. Maximal secretion of the gonadotropin Luteinizing Hormone is supported by GLUT1 expression and activity, and GnRH-induced glycolysis is recapitulated in primary gonadotropes. GLUT1 expression increases in vivo during the GnRH-induced ovulatory LH surge and correlates with GnRHR. We conclude that the gonadotropes of the anterior pituitary sense glucose availability and integrate this status with input from the hypothalamus via GnRH receptor signaling to regulate reproductive hormone synthesis and secretion.

## Introduction

Reproduction in females is a demanding, energy-expensive process. In humans and other mammals, the hypothalamic–pituitary–gonadal (HPG) axis, the endocrine regulator of reproduction, must decipher energy status and nutrient availability to determine if energy requirements to support reproduction can be met. Reduced nutrient availability negatively impacts reproduction, especially in women with low BMI. Underweight women (BMI < 18.5) often experience disrupted menstrual cycles due to a persistence of ovarian dysfunction^1,2^. Animal models have demonstrated a causal relationship between low nutrient availability and reproduction. In rats, mild food deprivation delays puberty onset, and suppresses estrous cyclicity and ovulation^3^. Likewise, Intermittent fasting in female rats, which results in reduced blood glucose levels, suppresses luteinizing hormone (LH) and disrupts estrous cycles^4^. These studies indicate that low nutrient availability is sensed by the HPG-axis and results in reduced LH secretion. However, the endocrine, cellular, and molecular mechanisms that mediate the effects of nutrient availability on the reproductive axis remain unclear.

Gonadotropes, the endocrine cells responsible for secretion of the gonadotropins LH and follicle-stimulating hormone (FSH), are located in the highly vascularized anterior pituitary with direct access to circulating nutrients. A population of hypothalamic neurons secrete gonadotropin-releasing hormone (GnRH) in a pulsatile manner into the portal vasculature of the anterior pituitary where is elicits the secretion of LH and FSH from gonadotropes. In females, these pituitary hormones act on the ovaries to promote folliculogenesis, oogenesis, and steroidogenesis. Sex steroids from the ovaries regulate this axis through both positive and negative feedback mechanisms. Several studies implicate the gonadotrope as a point for nutrient regulation of the HPG-axis in addition to evidence that kisspeptin and GnRH neurons in the central nervous system regulate LH pulsatility in response to glucose availability^5–8^. In normal women and in women with polycystic ovary syndrome, BMI negatively correlates with circulating LH^9^. In PCOS, responsiveness to GnRH and LH pulse amplitude, but not LH pulse frequency are also correlated, implicating the pituitary as an integrator of metabolic status in the HPG-axis independent of hypothalamic input^10^. Manipulating plasma glucose concentrations in animals further suggests that gonadotropes sense glucose availability. Lowering blood glucose in postpartum cows with phlorizin inhibits LH pulse amplitude^11^. Conversely, glucose infusions in castrated male sheep that result in a fourfold increase in plasma glucose concentration increased both LH pulse amplitude and frequency^12^. In vitro*,* primary mouse gonadotropes are responsive to glucose availability and express high levels of glucose transporter 1 mRNA (GLUT1, encoded by the *Slc2a1* gene)^13^. GLUT1 protein and glucose uptake are both increased in LβT2 cells, a gonadotropic cell line, in response to chronic GnRH stimulation in vitro*,* and this coincides with established effects of GnRH such as increased LH secretion^14^. Additionally, GLUT1 protein is increased in gonadotropes during puberty in mice^31^. Together, these studies suggest that glucose transport is associated with LH secretion and that gonadotropes may sense glucose and adapt gonadotropin secretion in response to energy availability.

Cells take up glucose or other sugar molecules by facilitated diffusion through the glucose transporter (GLUT) proteins encoded by the solute carrier family 2 (*Slc2a)* genes. The human genome encodes 14 GLUT family proteins, while the mouse genome encodes 12. The sequences of GLUT proteins, especially GLUT1 and 4 are highly conserved across species, and these two have been intensely studied^15^. GLUT1 is constitutively expressed and is ubiquitous. GLUT1 is deemed responsible for the maintenance of basal glucose uptake and transport of glucose across the blood brain barrier. GLUT4 is regulated by insulin in insulin-sensitive tissues, especially muscle and fat. The lesser-studied GLUT3 is a high affinity glucose transporter that can have a large impact at low expression levels and is found in neurons. GLUT8 is linked to reproductive regulation via its expression in the testis and blastocysts and may be regulated by insulin^16–18^. Primary gonadotropes predominantly express *Glut1* mRNA^13,14^, and tonic GnRH stimulation increases GLUT1 protein expression in a gonadotrope cell line^14^, indicating that gonadotropes may adjust their metabolism and hormone production in a glucose-dependent manner. Here, we report that regulation of GLUT1 by GnRH and subsequent glycolysis is a process that supports maximal secretion of LH. Using a novel fluorescence activated cell sorting (FACS) approach to culture wild-type mouse gonadotropes, we demonstrate that this process also occurs directly in primary pituitary cells and is correlated with GnRHR expression.

## Results

### GnRH regulates GLUT1 in gonadotropes

There is evidence that a global metabolic response in gonadotropes is associated with GnRH stimulation and LH secretion. mRNA-seq was performed on sorted pituitaries from female mice in proestrus (the cycle stage in which the LH surge occurs) and diestrus (cycle stage with generally low LH)^19^. Our independent secondary analysis of those data revealed that genes related to cellular catabolism, and therefore generation of energy, were generally increased during proestrus in comparison to diestrus (Supplementary Fig. S1). These data demonstrate that in vivo physiological changes in LH secretion are likely tied to gonadotrope cellular metabolism and are responsive to changes in upstream GnRH secretion which regulates the LH surge^20^. mRNA-seq analysis of GnRH-treated LβT2 cells^21,22^, a mature C57BL/6 mouse female gonadotrope cell line^23^, indeed demonstrates that GnRH regulates genes associated with gonadotrope cellular metabolism (Supplementary Fig. S1). LβT2 cells are an excellent model for deciphering mechanisms of GnRH action that can be subsequently validated in vivo, including regulation of LH and FSH secretion by GnRH pulse frequency and amplitude^19,24–27^. The gene ontology analysis of mRNA-seq data from LβT2 cells indicating metabolism as the most enriched biological pathway in gonadotropes in response to GnRH corroborates the in vivo observation that metabolic genes are upregulated in gonadotropes during proestrus (Supplementary Fig. S1). These findings provide a strong rationale to assess the relationship of cellular metabolism to GnRH-induced secretion of LH from gonadotropes.

**Figure 1:**
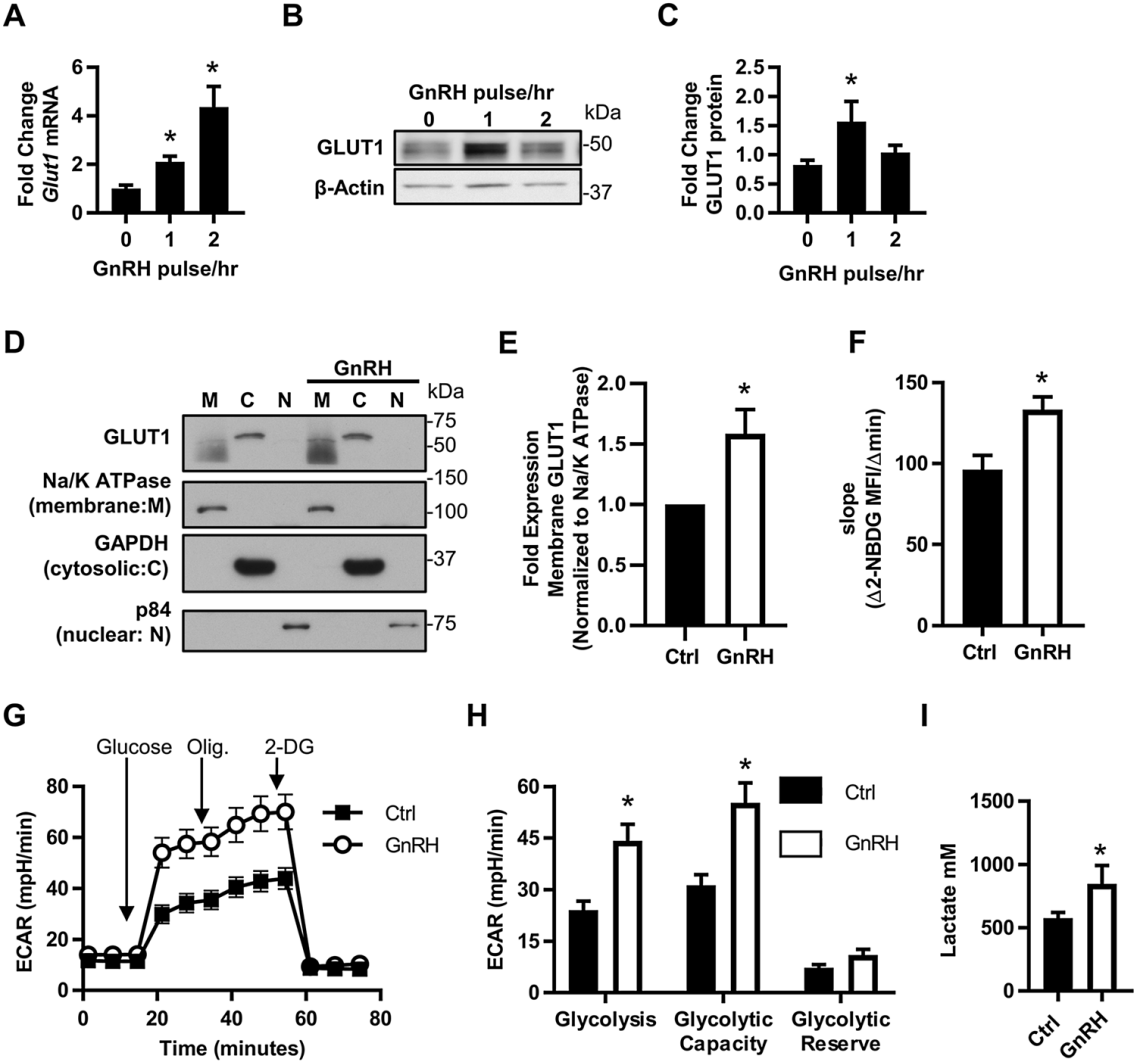
GLUT1 expression and activity is increased by GnRH. (**A**) mRNA and (**B**,**C**) protein expression of GLUT1 in response to increased frequency of GnRH pulsatility (N = 3). LβT2 cells were pulsed with 10 nM GnRH either once or twice per hour for 4 h. (**A**) Isolated RNA was analyzed by qRT-PCR, normalized to *Gapdh* and expressed as fold change to non-pulsed controls (0 pulse/h). (**B**,**C**) Protein lysates were assessed by western blot, quantified by chemiluminescent densitometry and normalized to β-Actin. Significance was determined by ANOVA and post hoc analysis with Dunnett’s comparison to control test. (**D**,**E**) LβT2 cells ± 30 min 10 nM GnRH were fractionated into cellular components and analyzed by western blot. Each experiment was run on one gel, the membrane cut, then probed simultaneously for all targets. GLUT1 in the membrane fractions was quantified by densitometry and normalized to Na^+^/K^+^ ATPase, then expressed as fold increase over control for each experiment (N = 5). Significance was determined by Student’s t-test. (**F**) 2-NBDG uptake in response to GnRH. The rate of 2-NBDG uptake was assessed by flow cytometry on LβT2 ± 30 min GnRH (N = 3). Significance was determined by Student’s t-test. All data are expressed as mean ± SEM. (**G**) A glycolytic stress test was performed on LβT2 cells. Presented is the average ECAR trace of three independent experiments performed with 10 technical replicates, N = 3. (**H**) Quantification of the glycolytic stress test in (**G**). Significance was determined by matched pairs analysis and Student’s t-test. (**I**) LβT2 cells were treated ± 10 nM GnRH after serum starvation for 30 min and lactate production was measured (four independent experiments N = 14). Significance was determined by Student’s t-test of Box Cox transformed values. See also Supplementary Figs. S1 and S2.

GnRH is secreted from hypothalamic neurons in a pulsatile manner, and GnRH pulse frequency and amplitude specifically regulate the downstream gonadotrope response. High frequency GnRH pulses favor LH production while low frequency GnRH pulses favor FSH production^25^. Similar to LH surge-associated genes, we hypothesized that increasing GnRH pulse frequency would increase *Slc2a1 mRNA*. To test this hypothesis, we analyzed *Slc2a1, Slc2a3, Slc2a4, and Slc2a8* mRNA expression data extracted from an mRNA array data set (GSE63251) of LβT2 cells pulsed with an amplitude of 10 or 100 nM GnRH at increasing frequencies^25^. These data showed that *Slc2a1* mRNA levels increase with frequency while having no impact on mRNA of other *Slc2a* family members (Table 1). To confirm that this observation is statistically significant in LβT2 cells, we pulsed these cells either once or twice per hour for 4 h with an amplitude of 10 nM GnRH. We found increased expression of *Slc2a1* mRNA in response to increased GnRH frequency (Fig. 1A). However, in contrast to chronic (non-pulsatile) 8 h GnRH treatment^14^, protein levels of GLUT1 were only mildly increased when pulsed once per hour, but not twice per hour (Fig. 1B,C, Supplementary Fig. S2). Because GLUT1 protein levels were only slightly impacted, we surmised that GnRH may be regulating GLUT1 protein translocation to the membrane in addition to gene transcription and translation. To test this possibility, we fractionated LβT2 cells after 30 min of 10 nM GnRH treatment and assessed GLUT1 protein by western blot. We found that GLUT1 in the membrane fraction of LβT2 cells was significantly increased after GnRH treatment (Fig. 1D,E, Supplementary Fig. S2). These data demonstrate that both GLUT1 expression and translocation are increased by physiological GnRH pulse stimulation.

**Table 1:** *Slc2a1,3,4*, and *8* mRNA expression in pulsed LβT2 cells.

Sample	Pulse per hour	Amplitude (nM GnRH)	Fold regulation			
			Slc2a1*^#^	*Slc2a3*	*Slc2a4*	*Slc2a8*
GSM1544462	1	10	4.77412	1.52463	0.69966	1.34887
GSM1544466	1	10	2.53663	2.56506	1.12612	1.28345
GSM1544461	4	10	6.88396	3.4666	1.01961	1.27408
GSM1544465	4	10	4.28798	1.25519	0.702	1.02983
GSM1544460	8	10	12.8469	2.93817	0.43011	1.24877
GSM1544464	8	10	12.5739	2.56071	0.36433	1.11692
GSM1544457	1	100	2.62153	3.80555	0.4576	0.12755
GSM1544471	1	100	4.68162	6.42664	0.79828	0.13867
GSM1544455	2	100	6.99814	3.72679	1.58625	0.03234
GSM1544469	2	100	14.7609	3.78888	0.45766	0.04163
GSM1544454	8	100	10.3734	5.901	0.5939	0.03207
GSM1544468	8	100	22.5277	4.24688	0.3451	0.0346

mRNA was isolated from LβT2 cells pulsed with an amplitude of 10 or 100 nM GnRH for 4 h and analyzed by RNA array^25^. Data were extracted from GEO dataset GSE63251 using GEO2R and are presented as fold change to 0 pulse control.

Significantly regulated by GnRH frequency* or amplitude^#^ as determined by Bonferroni MTC.

### Gonadotropes induce glycolysis in response to GnRH

The function of GLUT1 as a facilitative glucose transporter indicates that GnRH-induced GLUT1 at the cell surface will increase glucose uptake and therefore glycolysis. Using the fluorescent glucose analog 2-NBDG, we confirmed that a 30 min GnRH treatment induces a 1.4-fold increase in glucose uptake in LβT2 cells, similar to chronic GnRH treatment^14^ (Fig. 1F). We further tested the impact of GnRH on glycolysis with a glycolytic stress test via extracellular flux analysis which measures the extracellular acidification rate (ECAR, a proxy for acid production from all sources including lactate from glycolysis) in real time. We found that GnRH significantly increases both glycolysis and the maximum capacity of these gonadotropes to engage glycolysis (Fig. 1G,H). Because ECAR is only an indirect measure of glycolysis, we independently confirmed this result by measuring lactate (a product of anaerobic glycolysis) and found that GnRH increases lactate production by LβT2 cells (Fig. 1I). Together, these data demonstrate that gonadotropes rapidly respond to GnRH by increasing glycolysis.

### Glucose metabolism in gonadotropes is mediated by GLUT1

**Figure 2:**
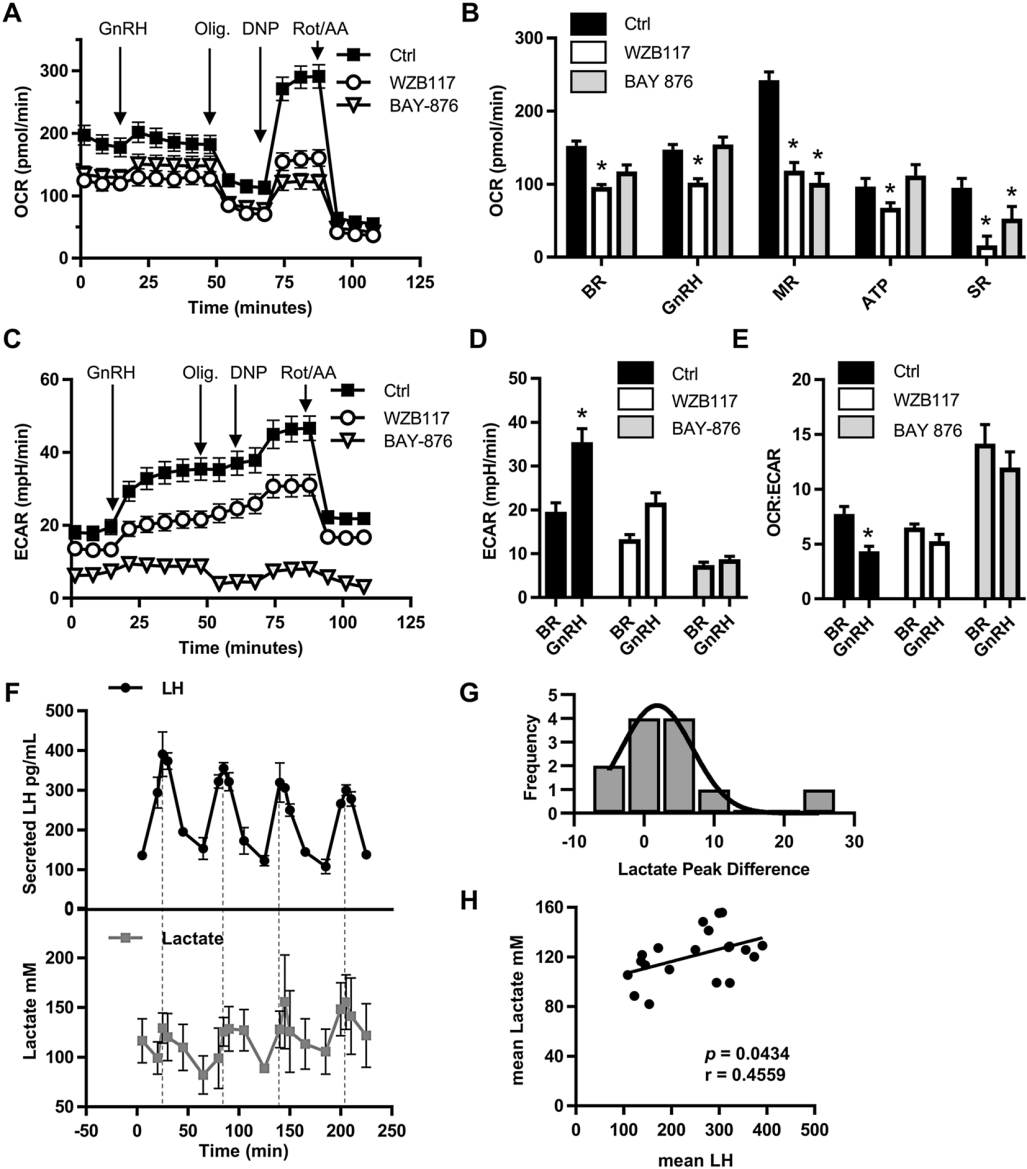
GLUT1 mediates GnRH-induced glycolysis in gonadotropes. (**A**,**C**) OCR (**A**) and ECAR (**C**) from mitochondrial stress test extracellular flux profiles for LβT2 cells ± GLUT1 pharmacological inhibitors, 10 μM WZB117 or 10 μM BAY-876 (three independent experiments performed with 10 technical replicates, N = 3). (**B**) Metabolic parameters derived from the LβT2 mitochondrial stress test profile in (**A**), 2-way ANOVA followed by post hoc analysis with Dunnett’s comparison to control test. (**D**) The basal and GnRH-induced ECAR in LβT2 cells derived from (**C**). Matched pairs analysis followed by Student’s t-test performed by inhibitor. (**E**) Basal and GnRH-induced OCR:ECAR ratio of LβT2 cells derived from (**A**,**C**), analyzed by ANOVA followed Studetn’s t-test for specific paired comparisons. (**F**) LβT2 cell cultures established on cytodex 3 microcarrier beads prepared as described in the Methods were loaded onto columns and perifused with serum-free DMEM. Columns were stimulated with hourly pulses of GnRH at peak values of ~ 10 nM for 4 h. Perifusate collected at 5 min intervals were analyzed and mean LH and lactate for 3 independent experiments were graphed. (N = 3). (**G**) Differences between LH and lactate peaks from (**F**) were evaluated using paired distribution analysis by nonparametric Wilcoxon signed rank testing of the difference in time of observed peak values. (**H**) Scatter plot of the mean LH value and mean lactate value from (**F**), N = 3 independent experiments. Significance determined by Spearman’s correlation.

Because GnRH induces GLUT1 translocation and glycolysis in gonadotropes (Fig. 1), we tested whether GLUT1 mediates GnRH-induced glycolysis. To examine glycolysis and to assess the potential contribution of mitochondria to GnRH-induced metabolic responses, we used extracellular flux (XF) analysis from a mitochondrial stress test to quantify oxygen consumption rate (OCR), a measure of mitochondrial respiration, and ECAR of LβT2 cells. Surprisingly, OCR remained the same in response to GnRH treatment, indicating that mitochondria do not energetically contribute to the GnRH response (Fig. 2A,B). In contrast, LβT2 ECAR increased and the OCR:ECAR ratio decreased in response to GnRH (Fig. 2C–E), suggesting that gonadotropes elevate glycolysis to support GnRH-induced activity. To determine whether GnRH-induced glycolysis is dependent on GLUT1, we performed a mitochondrial stress test on LβT2 cells with or without pharmacological inhibitors of GLUT1^28,29^. Both GLUT1 inhibitors WZB117 (IC_50_ = 10 μM) and BAY-876 (IC_50_ = 2 nM, selectivity factor > 100 against GLUT2, GLUT3, and GLUT4) significantly inhibited basal and maximal OCR and the spare respiratory capacity of LβT2 cells (Fig. 2A,B). Interestingly, only WZB117 reduced OCR and ATP production under GnRH-treated conditions (Fig. 2A,B). This may be due to the differential impact of these GLUT1 inhibitors on glycolysis in LβT2 cells. WZB117 inhibits GnRH-induced ECAR by ~ 40%, while BAY-876 inhibits basal and GnRH-induced ECAR by ~ 60% and ~ 80% respectively (Fig. 2C,D). Despite the near complete ablation of ECAR by Bay-876, LβT2 cells are able to maintain the majority of their OCR, indicating that GLUT1-mediated glycolysis contributes to, but is not necessary for mitochondrial respiration in these cells. Importantly, both GLUT1 inhibitors prevented a GnRH-induced switch to glycolysis as evidenced by the absence of decreased OCR:ECAR as seen in control LβT2 cells (Fig. 2E). From these data, we conclude that GLUT1 supports GnRH-induced glycolysis in gonadotropes.

Our observations demonstrate that a one-time physiological dose of GnRH induces glycolysis in LβT2 cells. In vivo, GnRH is pulsatile and elicits pulsatile secretion of LH^20^. A hallmark of gonadotropes is their ability to resolve signaling input from a GnRH pulse and reset for subsequent stimulation by a following pulse of GnRH^30,31^. Therefore, we examined whether GnRH-induced glycolysis is pulsatile with GnRH. We measured lactate and LH secretion from LβT2 cells in response to hourly GnRH pulses with an amplitude of 10 nM. Lactate peaks had a mean time difference of 3.75 min and a median difference of 2.5 min after LH peaks (Fig. 2F,G). Peak differences were not statistically different as determined by nonparametric testing and were within the 5-min fraction sampling interval (Fig. 2G). The transient increases in lactate associated with LH pulses suggest a temporary activation of glycolysis induced by GnRH. Additionally, the concentration of lactate positively correlated with mean secreted LH (Fig. 2H). These data implicate glycolysis in the regulation of LH secretion.

### Glucose supports maximal secretion of LH

**Figure 3:**
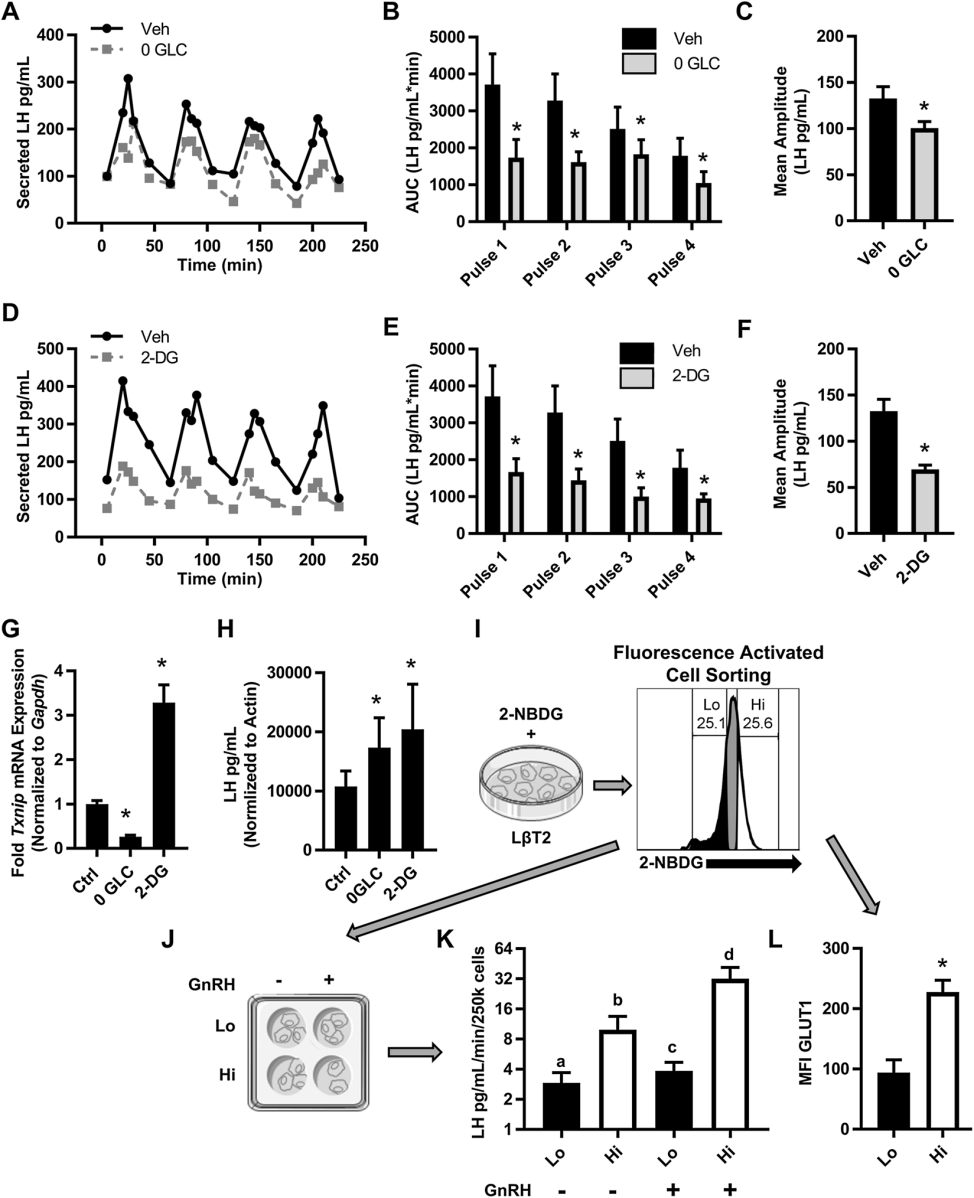
Glucose supports basal and GnRH-induced LH secretion. (**A**,**D**) LβT2 cell cultures established on cytodex 3 microcarrier beads were serum starved over night with vehicle, glucose-free, or 10 mM 2-DG media as described in Methods. These cells were loaded onto columns and perifused with serum-free DMEM. Columns were equilibrated with vehicle, glucose-free, or 10 mM 2-DG for 40 min prior to stimulation with hourly pulses of GnRH at peak values of ∼ 10 nM for 4 h. Perifusate collected in 5 min intervals were analyzed and graphed for (**A**,**D**) LH (Representative of N = 4). (**B**,**E**) Area under the curve (AUC) analysis of each pulse (represented in **A**,**D**). Significance was determined by ANOVA of Box Cox transformed data followed by Student's t-tests for specific paired comparisons. (**C**,**F**) Mean amplitude across all four pulses from (**A**,**D**), N = 4. Significance was determined by ANOVA of Box Cox transformed data followed by Student's t-tests for specific paired comparisons. (**G**,**H**) After pulse experiments (**A**–**F**) mRNA and total protein were extracted from harvested LβT2 cells. (**G**) Quantitative PCR of *Txnip* on pulsed LβT2 cells mRNA. (**H**) LH content in protein lysates of pulsed LβT2 cells. Results were analyzed by ANOVA followed by Student's t-tests for specific paired comparisons. (**I**) LβT2 cells were incubated with 2-NBDG then FAC sorted based on low (Lo) or high (Hi) fluorescence of 2-NBDG (three independent experiments). (**J**) After overnight in vitro culture and serum starvation, Lo and Hi sorted populations were stimulated with 10 nM GnRH for 30 min. (**K**) The conditioned media from (**J**) was assessed for LH (Basal, N = 6, GnRH, N = 3). Significance was determined by 2-way ANOVA on Box Cox transformed data. (**L**) LβT2 cells incubated with 2-NBDG were co-stained with anti-GLUT1 Alexa 647 and analyzed by flow cytometry. The mean fluorescence intensity (MFI) of GLUT1 Alexa 647 was determined for cells with Lo and Hi 2-NBDG fluorescence. Significance was determined by ANOVA and post hoc testing with Student’s t-test of specific pairs (N = 4).

Because GnRH-induces lactate production in gonadotropes that correlates with LH secretion, we expect that glycolysis is the process that supports energy-dependent LH transcription, translation, and secretion^26^. Therefore, we used two approaches to test the impact of glucose on pulsatile LH secretion from LβT2 cells. First, we performed pulse experiments with glucose-free media supplemented with pyruvate. The absence of glucose caused a significant reduction in total GnRH-induced LH secretion as quantified by the area under the curve and a ~ 30% decrease in mean LH amplitude (Fig. 3A–C). Second, we performed pulse experiments with media containing 2-DG, a glucose analog which competitively inhibits the production of glucose-6-phosphate from glucose and depletes cellular ATP. We observed a significant inhibition of GnRH induced-LH secretion across all pulses and a ~ 50% reduction in mean amplitude (Fig. 3D–F). To confirm that glucose metabolism in glucose-free conditions was reduced throughout the 4-h pulse experiment, we assessed expression of a known glucose responsive gene, thioredoxin interacting protein (*Txnip)*^32,33^, in cells at the completion of the experiment. As expected, LβT2 cells in glucose-free media have significantly reduced *Txnip* levels compared to control (Fig. 3G). Conversely, cells treated with 2-DG had a threefold increase in *Txnip*, reflecting the high presence of glucose available in the cells that cannot be metabolized due to the inhibition of glycolysis (Fig. 3G)^32,34,35^. To determine whether the reduction of LH secretion was due to reduced secretion or reduced biosynthesis, we determined LH content of LβT2 cells post pulse. We found that both glucose-free and 2-DG conditions resulted in increased LH stores (Fig. 3H). These data suggest that glucose supports maximal LH secretion. However, it remains unclear whether it is glucose availability or the level of glucose uptake that is important for regulation of LH synthesis and secretion.

To resolve the contribution of glucose availability versus glucose uptake, we leveraged the intrinsic heterogeneity of the LβT2 cell line^36^. Based on data showing that LH pulses are correlated with glycolytic bursts (Fig. 2F,G), we hypothesized that cells that take up more glucose will secrete increased amounts of LH. To test this hypothesis, we labeled LβT2 cells with 2-NBDG, a fluorescent glucose analog, and FAC sorted the cell line based on low glucose uptake (Lo, the 1st quartile) or high glucose uptake (Hi, the 4th quartile) (Fig. 3I). These cells were placed back into independent culture and treated ± GnRH for 30 min (Fig. 3J), after which secreted LH was measured (Fig. 3K). Both low and high glucose utilizers responded to GnRH with a significant increase in LH secretion (Fig. 3K). Impressively, high glucose utilizers secreted more basal LH than GnRH-stimulated low glucose utilizers. Coupled with the demonstration that high glucose utilizers had approximately twice as much surface GLUT1 as low glucose utilizers (Fig. 3L), this finding indicates that GLUT1 activity facilitates gonadotrope demand for glucose uptake during GnRH-induced LH secretion.

### Glucose support of LH secretion is facilitated by GLUT1

**Figure 4:**
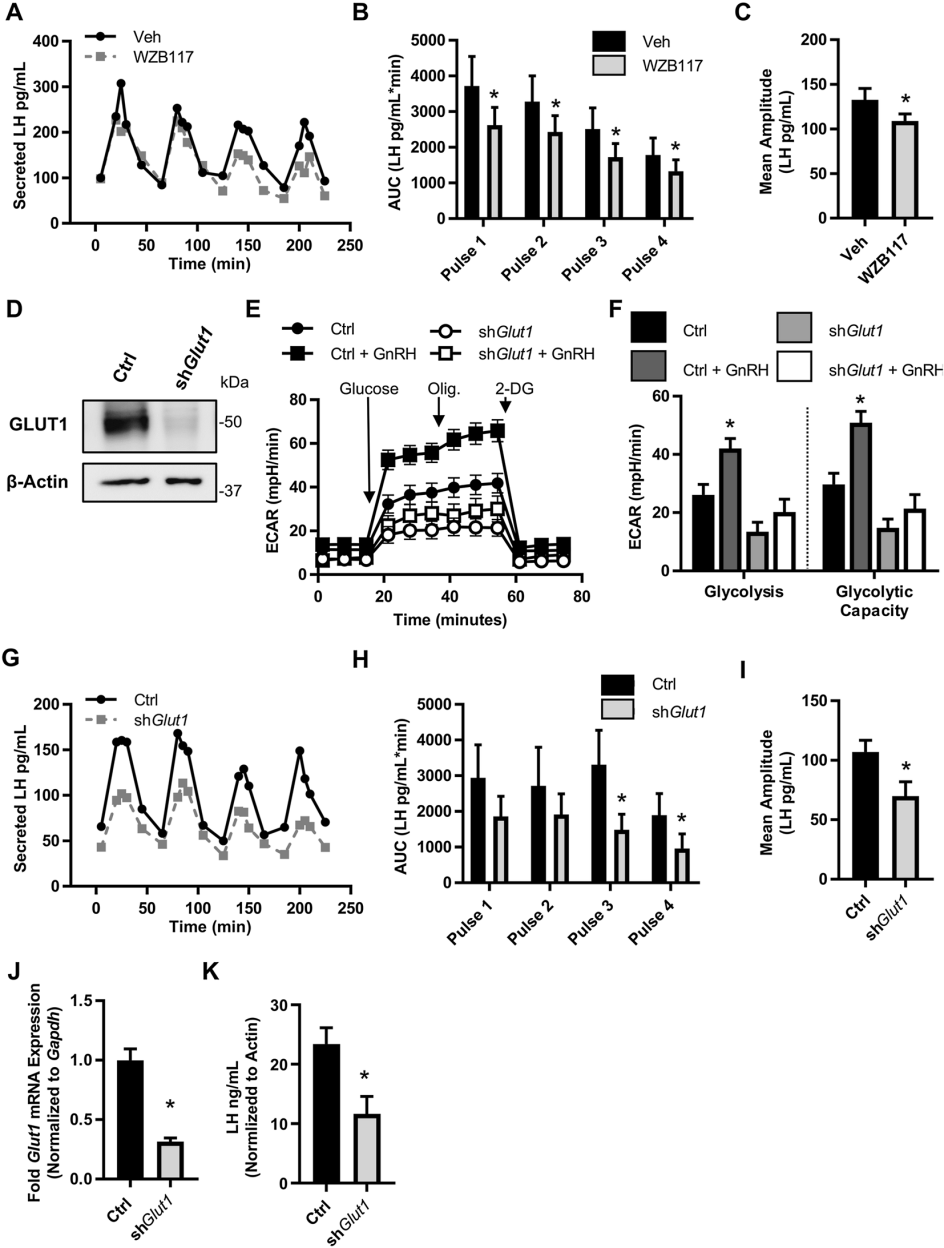
GLUT1 mediated glycolysis supports maximal GnRH-induced LH secretion. (**A**) LβT2 cell cultures established on microcarrier beads were serum starved overnight with vehicle or 10 μM WZB117. These cells were loaded onto columns and perifused with serum-free DMEM. Columns were equilibrated with vehicle or 10 μM WZB117 for 40 min prior to stimulation with hourly pulses of GnRH at peak values of ∼ 10 nM for 4 h. Perifusate collected in 5 min intervals were analyzed and graphed for (**A**) LH (Representative of N = 4). (**B**) Area under the curve (AUC) analysis of each pulse (represented in **A**). Significance was determined by Student’s t-tests on Box Cox transformed data. (**C**) Mean amplitude across all four pulses from (**A**), N = 4. Significance was determined by Student’s t-tests on Box Cox transformed data. (**D**) Western blot of GLUT1 knockdown in sh*Glut1* compared to Ctrl (non-targeting shRNA) transduced LβT2 cell lines. (**E**) A glycolytic stress test was performed on LβT2 cell lines. Presented is the average ECAR trace of three independent experiments performed with 10 technical replicates, N = 3. (**F**) Quantification of the glycolytic stress test in (**E**). Significance was determined by ANOVA followed by Dunnett’s comparison to control test on Box Cox transformed values. (**G**) LβT2 cell lines established on microcarrier beads were serum starved overnight. These cells were loaded onto columns and perifused with serum-free DMEM. Columns were equilibrated 40 min prior to stimulation with hourly pulses of GnRH at peak values of ∼ 10 nM for 4 h. Perifusate collected in 5 min intervals were analyzed and graphed for (**A**) LH (Representative of N = 3). (**H**) Area under the curve (AUC) analysis of each pulse (represented in **G**). Significance was determined by ANOVA on Box Cox transformed data followed by Student’s t-test of specific pairs. (**I**) Mean amplitude across all four pulses from (**G**) N = 3. Significance was determined by Student’s t-test on Box Cox transformed data. (**J**,**K**) After pulse experiments (**G**–**I**) LβT2 cells samples were divided in half and mRNA and total protein were extracted from paired samples. (**J**) qPCR of *Slc2a1* on mRNA from pulsed LβT2 cell line. (**K**) LH content in protein lysates of pulsed LβT2 cell lines. LH in protein lysates was assessed by Luminex and normalized to β-actin densitometry from equi-volume samples run on a western blot. See also Supplementary Fig. S1.

To test the impact of GLUT1-mediated glucose uptake on LH secretion, we inhibited the transporter with a pharmacological and molecular approach. First, we assessed pulsatile LH secretion from LβT2 cells in the presence of the GLUT1 inhibitor WZB117. Both total GnRH-induced LH secretion and the mean amplitude were significantly reduced in the presence of WZB117 (Fig. 4A–C). Second, we created stably transduced LβT2 cell lines expressing non-targeted shRNA (Ctrl) or shRNA targeted to *Slc2a1* (sh*Glut1*). The sh*Glut1* cell line exhibited ~ 70% reduction in both *Slc2a1* mRNA and GLUT1 protein (Fig. 4D, Supplementary Fig. S2). The sh*Glut1* cell line maintained normal signaling responses to GnRH as measured by immediate early gene induction by GnRH (*Egr1* and *Fos*) (Supplementary Fig. S2). Importantly, the knockdown of GLUT1 did not result in compensation by overexpression of other *Slc2a* family mRNA (Supplementary Fig. S2) but did recapitulate the ablation of GnRH-induced glycolysis observed with GLUT1 inhibitors (Fig. 4E,F). When we pulsed the Ctrl and sh*Glut1* cell lines with GnRH, we observed significantly lower LH secretion from the sh*Glut1* cells as compared to Ctrl (Fig. 4G–I). To ensure that the decrease in LH secretion from the sh*Glut1* cell line was due to reduced expression of *Glut1* and therefore less glucose uptake, we performed qPCR post-pulse to measure *Glut1* and *Txnip* mRNA . We found that the *Glut1* knockdown persisted throughout the pulse experiment (Fig. 4J) and that the sh*Glut1* cell line had significantly reduced glucose uptake, even less than WZB117 treated cells, as indicated by lower levels of *Txnip* expression (Supplementary Fig. S2). In contrast to the shorter-term approaches of glucose starvation and 2-DG treatment (Fig. 3H), the stable knock down of GLUT1 resulted in significantly lower LH content than control cells (Fig. 4K), indicating reduced LH synthesis may contribute to the reduced LH secretion in these cells. Together, these data support the conclusion that glucose uptake facilitated through GLUT1, though not necessary for basal or GnRH-induced LH secretion, supports maximal output of LH by gonadotropes.

### Primary mouse gonadotropes recapitulate GnRH-induced glycolytic switch observed in LβT2 cells

**Figure 5:**
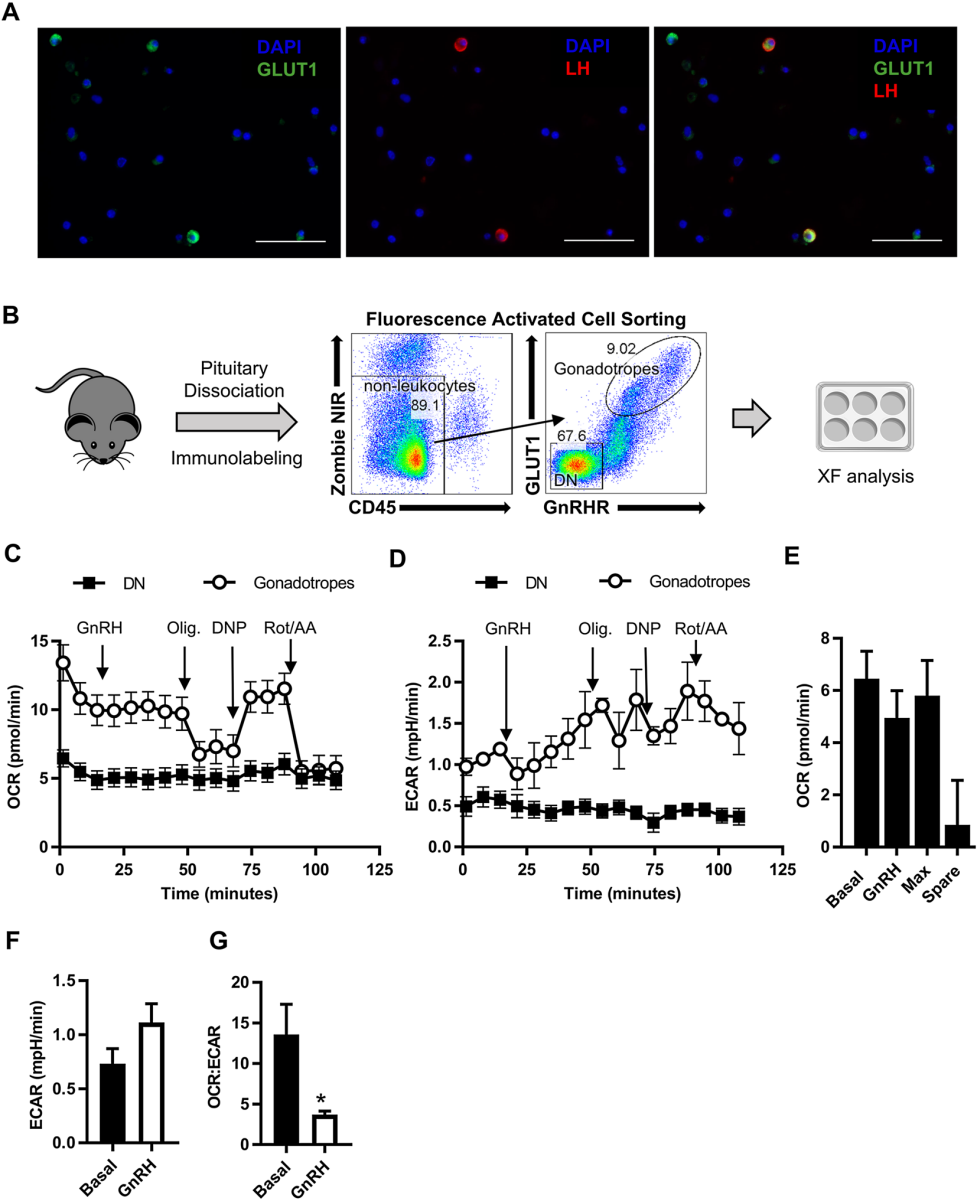
GnRH induces glycolysis in purified primary gonadotropes. (**A**) Primary pituitary cells cultured from 9- to 10-week-old female mice were fixed and stained with anti-GLUT1 antibody and anti-LH and visualized by secondary staining with Alexa Fluor 488–conjugated secondary antibody (green) and Alexa Fluor 568-conjugated (red) secondary antibodies, respectively. Nuclei were visualized with DAPI staining (blue). Scale bars, 50 µm. (**B**) Approach and flow cytometry gating strategy for sorting purified primary gonadotropes. Live non-leukocytes were gated as Zombie NIR viability dye negative and CD45 negative cells. Gonadotropes were sorted by positive expression of GnRHR and high expression of GLUT1. Cells that were negative for both GLUT1 and GnRHR were sorted as the double negative (DN) population. (**C**,**D**) OCR (**C**) and ECAR (**D**) from mitochondrial stress test extracellular flux profiles for sorted gonadotropes and double negative (DN) cells, 500,000 cells per well (N = 6). (**E**) Metabolic parameters derived from the gonadotrope mitochondrial stress test profile in (**C**). (**F**) Basal and GnRH induced ECAR derived from the gonadotrope ECAR profile in (**D**). (**G**) The basal and GnRH induced OCR:ECAR ratio derived from the gonadotrope mitochondrial stress test profile in (**C**). Significance was determined by Student’s t-test. See also Supplementary Figs. S3 and S4.

Our data in the LβT2 cell line provide evidence that gonadotropes sense glucose and can integrate this information to alter hormone production. Although primary gonadotropes are glucose responsive^13^, to date, no functional protein or metabolic assays have been performed on primary gonadotropes to verify glucose sensing. To answer the question of whether primary gonadotropes respond to GnRH by inducing glycolysis, we devised a protocol to obtain and culture purified primary gonadotropes, a prerequisite for XF analysis. High GLUT1 surface protein expression^37^, but not mRNA expression^38^, is exclusive to gonadotropes in the pituitary. Using immunocytochemistry (ICC) on dispersed primary pituitary cultures, we confirmed this observation by demonstrating that bright GLUT1 staining almost exclusively colocalizes with LH positive cells (Fig. 5A). Leveraging this pattern of GLUT1 expression in gonadotropes, we developed a FACS staining workflow to isolate gonadotropes from wild-type mice based on the co-expression of GLUT1 and the GnRH Receptor (GnRHR), exclusive to gonadotropes (Fig. 5B, Supplementary Fig. S3). This method captures sufficient cell numbers for XF analysis and eliminates the need for transgenic overexpression of fluorescent proteins, which introduce metabolic artifacts^39^. We validated that this sorting approach yielded viable purified primary gonadotropes. As expected, gonadotropes made up to 10% of pituitary cells with females having ~ 2% more gonadotropes than male mice (Supplementary Fig. S4). Cells co-expressing GLUT1 and GnRHR secreted basal LH and responded to GnRH in vitro by increasing secretion of LH, while sorted double negative cells (DN) did not (Supplementary Fig. S4). Lastly, we determined that the sorted gonadotropes were > 99% pure as measured by the percent of cells staining positive for LH by ICC post sort (Supplementary Fig. S3).

Using primary gonadotropes (GLUT1^hi^GnRHR^+^) and non-gonadotropes (DN, double negative) in a mitochondrial stress test, we found that gonadotropes are more bioenergetically active than other cells of the pituitary (Fig. 5C,D). In response to GnRH, we observed no change in OCR, just as in the LβT2 cell line (Fig. 5C,E). Further analysis of primary gonadotrope energetics revealed they have little to no spare respiratory capacity (the difference between basal and maximal respiration), indicating that any increase in metabolism must occur outside of the mitochondria (Fig. 5E) (i.e., additional ATP demand in gonadotropes must be met by glycolysis). We found that GnRH treatment appeared to increase ECAR (Fig. 5F), but more importantly, GnRH treatment reduced the OCR:ECAR ratio (Fig. 5G). These data demonstrate that primary gonadotropes, like LβT2 cells, switch from mitochondria-dependent metabolism to glycolysis in response to GnRH (Fig. 5C–G).

### Glucose supports basal LH secretion from primary mouse pituitary cultures

**Figure 6:**
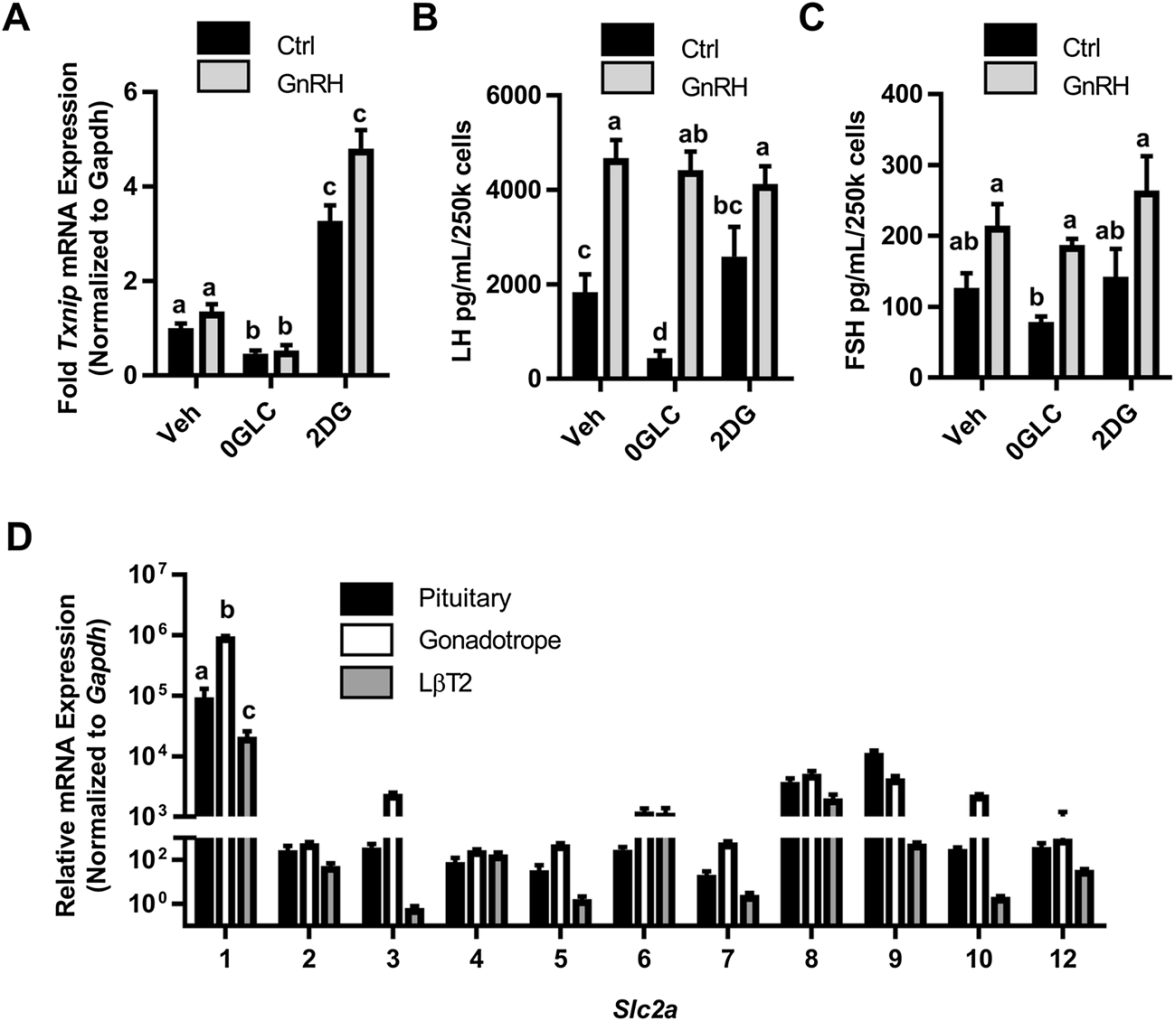
Glucose supports basal LH secretion from primary female pituitary cells. (**A**–**C**) After overnight in vitro culture and serum starvation in control, glucose-free, or 10 mM 2-DG conditions, dispersed primary pituitaries were stimulated with 10 nM GnRH for 30 min. qPCR for *Txnip* was performed on mRNA isolated from these cells (**A**), and conditioned media from the cultures was assessed for LH (**B**) and FSH (**C**). Significance was determined by ANOVA followed by post hoc testing using the Tukey HSD multiple comparison test. (**D**) Each member of the *Slc2a *family of genes was assessed by qRT-PCR performed on RNA from unstaged female whole pituitary, sorted gonadotropes, and LβT2 cells (N = 3, each primary sample pooled from a minimum of three mice). Significance was determined by ANOVA followed by post hoc testing using the Tukey HSD multiple comparison test.

To test whether blocking glycolysis has a similar impact on LH and FSH secretion from primary cells as it does on LβT2 cell LH secretion, we measured secreted LH from mixed primary pituitary cultures, not purified gonadotropes, in control, glucose-free, or 2-DG conditions. After confirming with *Txnip* expression that glucose-free and 2-DG conditions impacted primary cells similar to the LβT2 cell line (Fig. 6A), we found that primary pituitary cells have significantly reduced secretion of basal LH, but not FSH in glucose-free conditions (Fig. 6B,C). Surprisingly, LH and FSH secretion were not impacted by 2-DG treatment, highlighting a potential difference of ATP production and energy utilization between primary cells and LβT2 cells.

GLUT1 is only one of a family of glucose transporters that may facilitate glucose uptake in primary gonadotropes. Using mRNA isolated from whole pituitary, primary sorted gonadotropes (Fig. 5B) and LβT2 cells, we performed expression profiling of *Slc2a* 1–10, and 12 mRNA and demonstrate that like LβT2 cells, *Slc2a1* is the most highly expressed glucose transporter in primary gonadotropes in female mice (Fig. 6D and ^13^). Importantly, we found that primary gonadotropes exhibit the same *Slc2a* expression profile as LβT2 cells, though at a higher level. These data suggest that GLUT1 is the main glucose transporter in primary gonadotropes, just as in LβT2 cells. Additionally, expression of *Slc2a1* is an order of magnitude higher in purified gonadotropes than in whole pituitary in which they comprise approximately 10% of the population of cells, supporting the finding that GLUT1 expression in the pituitary is predominantly from gonadotropes.

### GLUT1 expression in the pituitary correlates with GnRHR

**Figure 7:**
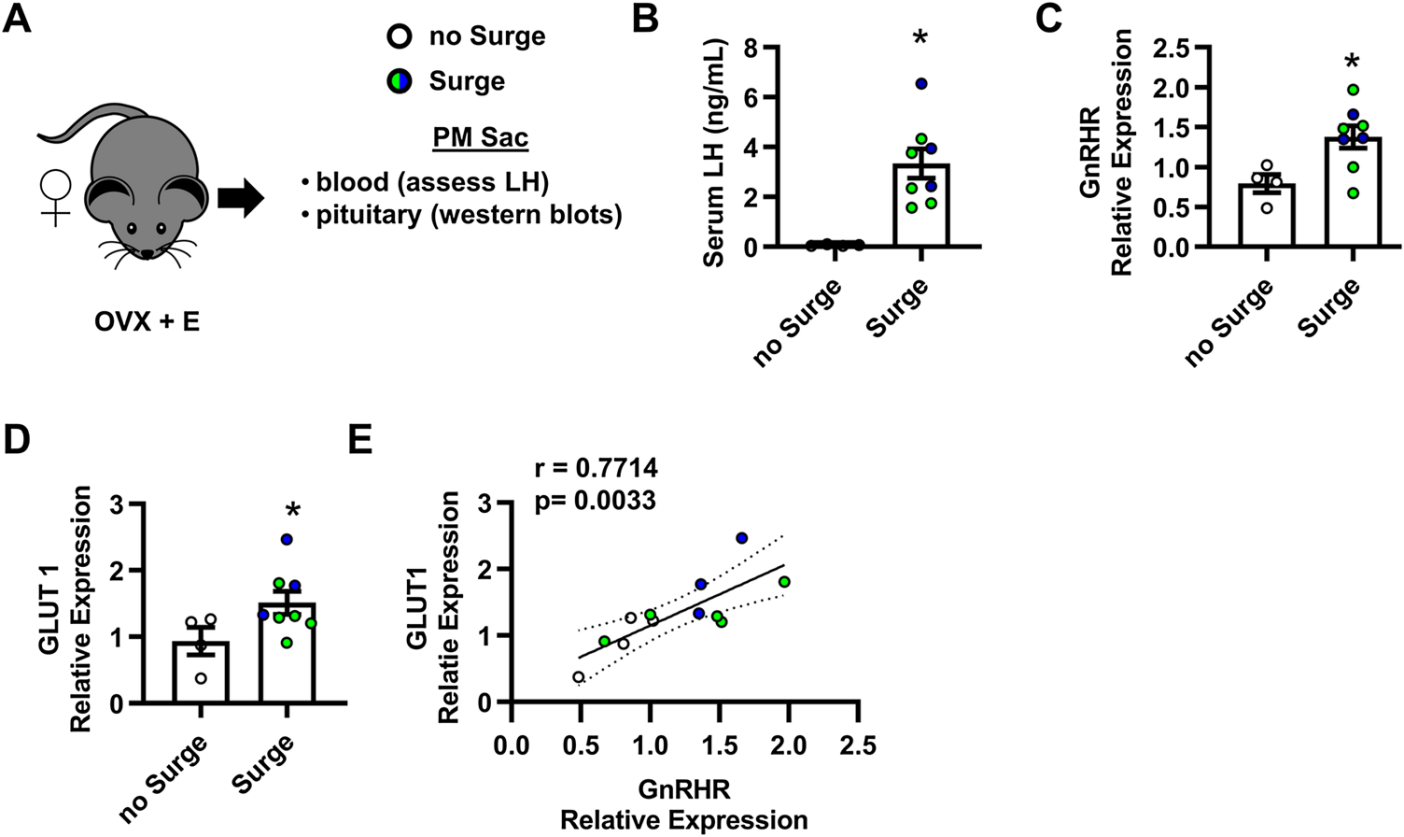
GLUT1 correlates with the LH surge in female mice. (**A**,**B**) The LH surge was induced in female mice by ovariectomy and estrogen (**E**) treatment. Pituitary and blood (serum) were collected on the evening of the surge. Serum LH concentrations < 0.6 ng/ml is designated as no Surge, N = 4. Concentrations > 1.5 ng/ml is Surge, N = 8 (**B**). For subsequent panels, hollow circles = no Surge, blue circles = Surge induced by estrogen pellets, and green circles = Surge induced by estrogen injections, both as detailed in the methods. (**C**,**D**) Quantification of western blot analysis of protein lysates from pituitaries (**A**,**B**). Significance in (**C**,**D**) was determined by Student’s t-test on Box Cox transformed values. (**E**) Correlation between GLUT1 and GnRHR protein expression performed on Box Cox transformed values. The number in the top left is the Pearson correlation coefficient (r). Shaded is the 95% fit confidence region. Pearson r and p values without the blue points (Surge induced by estrogen pellets) = 0.6883 and 0.0404, respectively. See also Supplementary Fig. S5.

The ability of GLUT1 to facilitate maximal GnRH-induced LH secretion (Fig. 4) implies that expression of this transporter may be important for LH secretion in vivo. The LH surge is an acute rise of LH to concentrations higher than any other time of the estrous cycle and is the trigger for ovulation in female mice. GnRH binding to gonadotropes increases prior to the LH surge and declines thereafter^40–42^. If the expression of glucose transporters is indeed important for the LH surge, we expect that GLUT1 will be increased in the pituitary with the LH surge and will correlate with GnRHR expression. To test our hypothesis, we ovariectomized female mice and treated them with a well-established estrogen protocol to induce an LH surge^43–45^. As expected with this LH surge paradigm, approximately 2/3 of the mice treated with estrogen exhibit an LH surge as measured by serum LH (Fig. 7A,B). Partitioning the data by mice with and without a surge, we found that GnRH receptor protein expression in the pituitary is increased with the LH surge when pituitaries are collected at 6 PM, or lights out on the day of proestrus (Fig. 7C, Supplementary Fig. S5). We also found that GLUT1 protein expression in the pituitary is increased (Fig. 7D, Supplementary Fig. S5). Interestingly, these data mirror the impact of increasing frequency or amplitude of GnRH pulses on mRNA expression of *Slc2a1* (Table 1) in our initial observations in LβT2 cells. Next, we combined the data from all mice and performed correlation analysis of protein expression of GnRHR and GLUT1. Our analysis found that GLUT1 positively correlates with GnRHR (Fig. 7E). These data further support the importance of GLUT1 in gonadotrope function. We conclude that GLUT1 expression in gonadotropes correlates with GnRHR expression.

## Discussion

In this study, we demonstrated that GLUT1 facilitates glucose uptake and glycolysis in support of LH secretion from gonadotropes. Further, we provide evidence to support investigation of gonadotrope GLUT1-mediated glucose metabolism in facilitating the LH surge. These findings implicate gonadotropes in the overall response of the HPG axis to alterations in energy balance such as hypoglycemia or hyperglycemia.

Our data show that GnRH induces translocation of GLUT1 to the cell membrane, which is important for gonadotrope function. This work extends the established central role of GLUT1 in CD4^+^ T cell activation to another mammalian system^46^, namely gonadotrope control of reproduction. Though the mechanisms of GnRH regulation of GLUT1 translocation are to be determined, we have revealed a potential gonadotrope-specific regulation of GLUT1. GLUT1 expression and localization at the cellular membrane is classically regulated by local glucose concentration through glucose responsive mechanisms^47^. Low glucose availability induces translocation of GLUT1 to the surface to increase cellular glucose uptake while high glucose availability causes the converse through several sensors of glucose metabolites including carbohydrate response element–binding protein (ChREBP), sirtuin 1 (SIRT1), and AMP-dependent protein kinase (AMPK)^47^. Specifically, AMPK blocks the internalization of GLUT1 by phosphorylating and degrading TXNIP protein, which induces GLUT1 endocytosis, thereby increasing glucose uptake^32^. Because AMPK is a known signaling intermediate in GnRH-induced activation of *Lhb* transcription, GnRH degradation of TXNIP protein through activation of AMPK is a possible mechanism by which GnRHR signaling results in increased GLUT1 at the plasma membrane and increased glucose uptake^48^.

The ability of GnRH to induce GLUT1 translocation closely ties the regulation of glycolysis to regulation of LH secretion. There are several potential reasons why glucose metabolism may be important for LH synthesis and/or secretion. (1) ATP from glycolysis could support energy-dependent biosynthesis in response to GnRH stimulation. Deriving ATP from glycolysis is an accessible mechanism to control ATP availability to meet acute demand in a pulsatile manner through GnRH-controlled cycling of GLUT1. Gonadotropes express ATP-gated purinergic (P2X2R) Ca^2+^ channels^49^. Extracellular ATP induces a transient rise in cytosolic Ca^2+^ that stimulates LH release, and GnRH signaling in primary pituitary cultures increases ATP release, potentially activating parallel autocrine signaling that amplifies LH release^50,51^. Though we have not measured the impact of GLUT1 on ATP release from gonadotropes, other work implicates increased glycolysis as a potential source of autocrine ATP activation of P2X2Rs to support increased LH secretion. (2) Another attractive mechanism is the necessity of ATP to fuel parallel GnRH-induced activation of the cAMP pathway^52^. The cAMP/protein kinase A (PKA) pathway enhances LH release and increases the expression of LHβ and GnRHR^53,54^. GnRHR signaling was shown to be coupled to the cAMP pathway in LβT2 cells^55^ and in αT3-1 cells, an immature gonadotrope cell line^52^. During the preovulatory surge in female rats, there is a concurrent accumulation of cAMP in the pituitary which can be blocked with a GnRH antagonist, ganirelix^56,57^. Our data show that GLUT1 expression is increased during the LH surge and could be a source of ATP for the coincidental cAMP surge. Analysis of GnRH induced cAMP is warranted in the context of GLUT1 knockdown in LβT2 cells. (3) A third reason why glycolysis may be important for gonadotrope secretion of LH is the generation of nucleic acid precursors and maintenance of redox balance through the pentose phosphate pathway. Regulation of GLUT1 has been shown to suppress reactive oxygen species through its supply of the pentose phosphate pathway metabolite glucose-6-phosphate^58,59^. The pentose phosphate pathway is an important source of NADPH, an electron donor. NADPH protects against redox stress by providing reducing equivalents to antioxidants such as sulfiredoxin, which is necessary to resolve GnRH-induced oxidative stress^31^. In addition to needing reducing equivalents from NADPH, sulfiredoxin, a GnRH-induced redox factor, is also ATP dependent, further highlighting the integral role that glycolysis as an ATP source may play in the regulation of GnRHR signaling. Furthermore, the oxidation of NADPH provides reducing power for anabolism within cells including protein synthesis.

Mechanisms discovered in gonadotrope cell lines are often difficult to validate in primary cells due to the low representation of gonadotropes in the pituitary. Here, we used a FAC sorting approach that allowed us to perform functional studies (i.e. protein and metabolic analysis) on purified gonadotrope cell cultures. Our approach obviates the need for transgenic mice and will make the study of primary gonadotropes broadly accessible. Interestingly, the clear difference in surface protein expression of GLUT1 on gonadotropes compared to other cells of the pituitary is not seen on the mRNA level^38^. In fact, *Slc2a1* is ubiquitously expressed throughout the pituitary, and within the gonadotrope population *Slc2a1* expression varies. This difference in protein and mRNA expression point to post-transcriptional regulation of *Slc2a1* within the pituitary that needs further investigation. Gonadotropes may utilize RNA binding proteins to post-translationally regulate GLUT1 expression. We have previously demonstrated that the RNA binding protein ELAV-like protein 1 (ELAVL1) is important for the stabilization of gonadotrope mRNA including *Gnrhr*^60^. ELAVL1 stabilizes mRNA and regulates translation of *Slc2a1* in 3T3-L1 cells. Data from Terasaka et al. demonstrates that *Slc2a1* mRNA is also bound to ELAVL1 in gonadotropes^60^. These data suggest that discrepancies in protein and mRNA expression of GLUT1 may be due to translation control. It remains to be determined whether the surface co-expression of GLUT1 and GnRHR can be translated to other mammalian species. However, acute glucose sensitivity is a common feature of the mammalian HPG axis. In comparison to the LβT2 cell line, our studies in primary mouse gonadotropes revealed differences in the contribution of glucose metabolism to LH secretion. Unlike LβT2 cells, primary gonadotropes are not dividing and have a substantially higher resting OCR:ECAR ratio than LβT2 cells. This indicates that primary gonadotropes may have a higher capacity to rescue LH secretion in glucose-free conditions by relying on mitochondria. Subplasmalemmal mitochondria in gonadotropes contribute to GnRH-induced reactive oxygen species and Ca^2+^ flux^61^, a mechanism which supports LH synthesis and secretion. Further, L-type calcium channels are closely associated with these sub-plasmalemmal mitochondria and appear coordinated with ROS generation by GnRH-induced NADPH oxidase activity^61^. This suggests overall that ion channel activation, GLUT1 activity, and ROS generation may be interconnected in eliciting LH secretion in response to GnRH receptor activation.

We observed that glucose-free conditions inhibited basal LH secretion from both LβT2 and primary cells, while GnRH-induced secretion was only inhibited in the cell line. Interestingly, we found no impact on FSH secretion by glucose-free conditions. These results extended the paradigm of differential regulation of LH and FSH at the level of transcription and secretion studied by many research groups including our own. In our recently published studies we show that FSH is indeed co-secreted with LH in primary mouse gonadotropes in response to pulsatile GnRH stimulation^62^. However, LH appears to be the most sensitive to regulation by GnRH and is impaired by the presence of oleate. Al-Safi et al. did show that, in normal weight women, polyunsaturated fatty acids suppress FSH^63^. This suggests, that FSH is not immune to alterations in energy balance, though this topic is not fully explored. One can speculate that FSH stimulation preserves the pool of developing follicles and is necessary for long-term viability of the oocyte reserve, while LH operates on a shorter time frame and promotes ovulation rather than maturation. These could be evolutionary pressures which resulted in differential sensitivity of gonadotropin secretion to energy balance.

We found that inhibition of glycolysis and ATP by 2-DG had no impact on LH secretion from primary pituitary cultures. This resistance to 2-DG could be due to several factors including the high OCR:ECAR ratio of primary gonadotropes, the support of other pituitary cells in the culture, or the fact that primary LH secretion was tested in response to a single treatment with GnRH as opposed to pulsatile stimulation. Mouse studies with conditional KO of GLUT1 in the gonadotrope to test the dependence of gonadotropes on GLUT1-mediated glucose metabolism will further our knowledge of how the gonadotrope interprets or adapts to energy status to regulate reproduction. Particularly, in vivo studies of gonadotrope GLUT1 KO will help define the role of GLUT1 in the LH surge.

A reduction in reproductive fitness as a physiological response to reduced energy has been long-established. Here we highlight a fundamental cellular and molecular mechanism which may help explain how energy availability can be interpreted by the HPG axis at the level of the pituitary. Though nutrient sensing is known to occur in the hypothalamus, this work makes evident a novel inherent characteristic of gonadotropes, namely regulation of LH secretion through GLUT1-mediated glucose uptake, that serves to integrate energy status signals with reproduction.

## Methods

### LβT2 gonadotrope cell culture

The female C57BL/6 mouse-derived LβT2 gonadotrope cell line^23,64^ was maintained in high-glucose (4.5 g/l) HEPES-buffered DMEM supplemented with penicillin/streptomycin and 10% fetal bovine serum (FBS: FB-11, Omega Scientific, CA) at 37 °C in a humidified atmosphere of 5% CO_2_. Lentivirus transduced LβT2 cells were maintained under the same conditions with puromycin (0.25 µg/ml)-supplemented DMEM. To test the effects of GnRH, LβT2 cells were seeded at 2 × 10^5^ cells per cm^2^ (unless otherwise noted), cultured for 24 h, and pretreated with serum-free DMEM for 12–16 h prior to hormone treatment.

### Primary mouse pituitary cell culture

Mouse experiments were performed in accordance with UCSD Institutional Animal Care and Use Committee regulations under an approved protocol. Whole pituitaries were dissected from wild-type C57BL/6 female mice at 9–10 weeks of age. Whole pituitaries were isolated into ice-cold PBS and then dispersed by incubation with 0.25% collagenase Type IV and 0.25% trypsin–EDTA (1x) (Life Technologies) as previously described^65^. The cells were plated on poly-l-lysine (Sigma-Aldrich Inc.) coated Nunc 96-well plates (Thermo Fisher Scientific) at a density of 1.5 × 10^6^ cells per cm^2^. The cells were cultured for 24 h at 37 °C and 5% CO_2_ in high-glucose HEPES-buffered DMEM with 10% FBS prior to experimentation.

### Lentivirus transduction and puromycin selection

Lentivirus used for knockdown of GLUT1 was prepared using the Lenti-X packaging system (Takara Bio USA, CA) with pLKO.1 plasmid. Non-targeting shRNA (Sigma-Aldrich, Mission SHC002) and GLUT1 shRNA (Sigma-Aldrich, Mission TRCN0000079328) encoding pLKO.1 plasmids were separately transfected into Lenti-X 293T cells (Takara Bio USA, CA) in complete DMEM with 10% FBS. Lentivirus-containing supernatants were collected 48 and 72 h after transfection, filtered through a 0.45 μM polyethersulfone syringe filter, and concentrated by 10% polyethylene glycol 8,000 incubation for 16 h followed by centrifugation at 1,600 RCF for 1 h at 4 °C. Viral particles were resuspended in 1/10 of the original volume in serum and antibiotic free-DMEM. Lenti-X GoStix (Takara Bio USA, CA), a rapid test to determine p24 levels in supernatant preparations, were used to determine the viral titer. LβT2 cells were cultured in 24-well plates in complete DMEM with 10% FBS. On the next day, lentivirus was added in serial dilutions with 4 µg/ml polybrene (Fisher Scientific) and incubated for 4 h at 37 °C, followed by a change to DMEM with 10% FBS. As previously published^60^, the transduced cells were selected by culture in 0.5 µg/ml puromycin in DMEM with 10% FBS after 48 h incubation at 37 °C. The media with 0.5 µg/ml puromycin was changed every 2–3 days. Surviving cells were maintained in DMEM with 10% FBS supplemented with 0.25 µg/ml puromycin.

### Immunofluorescence

Mouse primary pituitary cells (2.5 × 10^5^ cells/cm^2^) or sorted gonadotropes (5.5 × 10^4^ cells/cm^2^) were seeded on poly-l-lysine-coated 4-well or collagen R (Fisher Scientific)-coated 16-well chamber slides (Lab-Tek) respectively and cultured in complete DMEM with 10% FBS overnight. After 8-h serum starvation, cells were fixed with 4% formaldehyde in PBS pH 7.4 on ice for 15 min, then washed. LH was detected using guinea pig antisera to rat LHβ polyclonal antibody (1:2,000) from the National Hormone and Peptide Program. Rabbit anti-GLUT1 monoclonal antibody (1:100) (Cell Signaling, Inc., Cat # 12939S, RRID: AB_2687899) was used to detect GLUT1. Goat anti-guinea pig Alexa Fluor 568 (Cat # A21450, RRID: AB_2735091) and goat anti-rabbit Alexa Fluor 488 (Cat # A11008, RRID: AB_143165) (both 1:800) (Life Technologies, CA) were used for secondary staining. Vectashield mounting medium with 4′, 6-diamidino-2-phenylindole (DAPI) (Life Technologies) was used to mount slides. Wide-field fluorescence images were captured using the NIS-Elements software (Nikon America Inc., Rochester, NY) on a Nikon TE2000-U microscope (Nikon America Inc., Melville, NY) equipped with an X-Cite 120PC collimated light source (Lumen Dynamics Group Inc., Ontario, Canada) and a CoolSNAP EZ monochrome camera (Photometrics Inc., Tucson, AZ) with DAPI-1160A, FITC-5050A-000 and mCherry-c filter sets (Semrock, Rochester, NY). The number of cells positive or negative for LH staining were manually counted.

### Glucose uptake by flow cytometry

LβT2 cells were grown in complete media (4.5 g/l glucose with sodium pyruvate) supplemented with antibiotics and 10% fetal bovine serum. Cells were harvested after a PBS wash with 0.25% trypsin, washed with PBS again and stained in PBS + Zombie NIR (Biolegend, Cat # 423106) diluted 1:1,600 for 20 min on ice protected from light. Cells were pelleted and resuspended in serum free DMEM (4.5 g/l glucose with sodium pyruvate), 1 × 10^6^ cells/200 μl/FACS tube. 10 nM GnRH was added to cells 30 min before flow cytometry analysis. Immediately prior to acquisition, 300 μM 2-(*N*-(7-Nitrobenz-2-oxa-1,3-diazol-4-yl)Amino)-2-Deoxyglucose (2-NBDG) (ThermoFisher Scientific) (2-NBDG) (Life Technologies, cat # N13195) was added to samples. Samples were acquired on slow for 1 min, followed by medium for 30 s, then high for 30 s to monitor that rate of increase in 2-NBDG fluorescence and ensure that the signal became saturated. The rate of glucose uptake was determined by the slope of the line produced by 2-NBDG mean fluorescence intensity plotted vs time (time gate = 15 s to 45 s). Samples were acquired in technical triplicates on a FACSCanto II flow cytometer (BD Biosciences, NJ, USA) and analyzed with FlowJo Software v 10.6.2 (Treestar, Ashland, OR, https://www.flowjo.com/).

### FAC sorting

To sort LβT2 based on glucose uptake, LβT2 in T75 flasks were incubated with 5 ml of glucose and pyruvate-free DMEM with 150 μM 2-NBDG (Life Technologies, cat # N13195) for 30 min at 37 °C. Cells (~ 15 × 10^6^) were washed with warm PBS, trypsinized, counted, and then resuspended in PBS containing Zombie NIR Live/Dead fixable stain (Biolegend, Cat # 423106) diluted 1:400. Samples were incubated for 20 min at 4 °C protected from light, washed with FACS buffer (0.1%BSA, 2 mM EDTA in 1 × PBS) and then sorted on a FACS Aria II (BD Biosciences, NJ, USA). The two-way sort was performed with an 85 μm nozzle. Unstained cells and cells single stained with 2-NBDG or Zombie NIR were used to set appropriate sorting gates. The gating hierarchy for the sort was (1) size, FSC-A vs SSC-A (2) doublet discrimination, SSC-w vs SSC-H and FSC-W vs FSC-H (3) living cells and (4) two-way sort on 2-NBDG fluorescence bottom 25% and top 25%. Cells were collected into 1 ml of 50% FBS and 50% DMEM + antibiotics in a 15 ml conical tube. Post sort, LβT2 cells were pelleted, and seeded in NUNC 24-well plates in 500 μl media/2.5 × 10^5^ cells/well. Cells equilibrated for 24 h before overnight serum starvation. After serum starvation (1% FBS), media was gently replaced with 500 μl control or 10 nM GnRH containing serum-free media for 30 min. This conditioned media was analyzed for LH via Luminex assay.

To sort primary gonadotrope cells, dissected pituitaries from non-staged female wildtype C57BL/6 mice were dispersed as described above. The number of mice per prep varied according to the number of cells needed for downstream application from 3 for staining validation or RNA isolation to 30 for XF analysis. Dispersed pituitary cells were counted then stained with 1:800 diluted Zombie NIR Live/Dead fixable stain (Biolegend, Cat # 423106) in 100 μl per 1 × 10^6^ cells for 20 min at 4 °C protected from light. Following a wash with FACS buffer, cells were blocked with TruStain FcX (anti-mouse CD16/32) antibody (Biolegend, Cat # 101319, RRID: AB_1574973) in FACS buffer and stained for 20 min at 4 °C protected from light with primary antibodies in 50 μl per 1 × 10^6^ cells. Cells were stained with rabbit anti-GLUT1 Alexa647 (Abcam, 1:50, Cat # ab195020, RRID # AB_2783877), rabbit anti-GnRHR (Proteintech, 1:200, Cat # 19950-1-AP, RRID: AB_10644155), and rat anti-CD45 PerCP-Cy5.5 (Biolegend, 1:25, Cat # 103132, RRID: AB_893340). Cells were washed again, then stained with donkey anti-rabbit BV421 (Biolegend, 1:100, Cat # 406410, RRID: AB_10897810) for 20 min at 4 °C protected from light. Finally, cells were washed and resuspended in FACS buffer at 20 × 10^6^ cells/ml. The two-way sort was performed with a 70 μm nozzle. Single stained Ultracomp eBeads and ArC Amine Reactive Compensation Beads (Thermofisher) were used for hard compensation on the BD FACSDiva Software v6.1.3 (https://www.bdbiosciences.com/en-us/instruments/research-instruments/research-software/flow-cytometry-acquisition/facsdiva-software). Unstained pituitary cells, cells stained with secondary antibody only (anti-rabbit BV421), cells stained only with Zombie NIR, and internal biological controls were used to set sort gates. Additional validation of antibodies and sort approach are presented in Supplemental Fig. S3. The gating hierarchy for the sort was (1) size, FSC-A vs SSC-A (2) doublet discrimination, SSC-w vs SSC-H and FSC-W vs FSC-H (3) living CD45- and (4) two-way sort on GLUT1^hi^GnRHR^+^ and GLUT1^-^GnRHR^-^. Cells were collected into 1 ml of 50% FBS and 50% DMEM + antibiotics in a 15 ml conical tube. Post sort, primary gonadotropes were pelleted and seeded for experiments.

### Extracellular flux analysis

For mitochondrial stress tests, LβT2 cells were seeded on collagen IV coated XF96 plates at 6 × 10^4^ cells per well in HEPES-buffered DMEM supplemented with 10% FBS and antibiotics as described above. Cells were cultured for 24 h then serum starved overnight. Prior to XF analysis LβT2 cells were washed twice with extracellular flux (XF) assay media (5 mM HEPES buffered-DMEM containing 10 mM glucose, 4 mM l-glutamine, and 1 mM sodium pyruvate). The media was changed to XF assay media for analysis. Primary sorted mouse gonadotropes and non-gonadotropes (DN, double negative) were washed in XF assay media immediately after cell sorting. Primary sorted cells were adhered to Corning Cell-Tak (Fisher Scientific) coated XF 96 plates at 5 × 10^5^ per well. Primary cells were equilibrated for 1 h before XF analysis. Oxygen consumption rate (OCR) and extracellular acidification rate (ECAR) were measured using a modified mitochondrial stress test procedure as follows: basal OCR followed by sequential addition of 10 nM GnRH, 2.5 μM oligomycin (Calbiochem), 500 μM 2,4 Dinotrophenol (DNP) (Sigma-Aldrich) and 1 μM rotenone + 1 μM antimycin A (Enzo) with the XF96 Extracellular Flux Analyzer (Seahorse Bioscience). All extracellular flux mito stress test data analysis was performed using the Seahorse Explorer (SHORE) Analysis program^66^.

For glycolytic (glyco) stress tests, LβT2 cells were seeded as described for mito stress tests. Prior to XF analysis, LβT2 cells were washed twice with glyco stress test (XF) assay media (5 mM HEPES buffered-DMEM containing, 4 mM L-glutamine and 1 mM sodium pyruvate). The media was changed to glyco stress test XF assay media for analysis. Glyco stress test was performed according to standard protocol on an XF96 Extracellular Flux Analyzer (Seahorse Bioscience, Agilent Technologies). Basal ECAR was measured followed by sequential addition of 10 mM glucose, 2.5 μM oligomycin (Calbiochem), and 50 mM 2-DG (Sigma-Aldrich). Data were extracted using the Wave software v2.6.1 (Seahorse Bioscience, https://www.agilent.com/en/products/cell-analysis/cell-analysis-software/data-analysis/wave-desktop-2-6) and manually analyzed.

### Lactate assay

Lactate production was measured in conditioned media after 30 min stimulation of LβT2 cells with 10 nM GnRH with the Lactate Colorimetric/Fluorometric Assay Kit (Biovision) according to the manufacturer’s protocol.

### Subcellular fractionation

LβT2 cells were washed twice with ice cold 1 × PBS and harvested in 1 × PBS. Cells were centrifuged for 5 min at 2,655 RCF at 4 °C, resuspended in 250 µl of 1 × hypotonic lysis buffer (20 mM HEPES, pH 7.4, 10 mM NaCl, 3 mM MgCl2, Roche protease cocktail inhibitors and 1 mM PMSF), and incubated for 30 min to allow for cell lysis. Cells were triturated using 10 passes through a 27 ½ G needle. After BCA quantification of the lysates, samples were diluted to protein concentration of the sample with the lowest protein concentration (2–2.5 mg/ml). The lysate was centrifuged at 2,655 RCF for 10 min and the pellet (nuclear “N”) was set aside. The supernatant (containing membrane “M” and cytosolic “C” proteins) was centrifuged again at 2,655 RCF for 10 min to pellet residual “N” fraction. The pellets from the two centrifugations were combined, washed twice, and resuspended with 250 µl of hypotonic buffer. The supernatant (“M” + “C”) was centrifuged (Rotor TLA120.2 Beckman Coulter Cat. # 343778) at 96,000 RCF for 1 h at 4 °C and the pellet (“M”) was set aside. The supernatant was again centrifuged at 96,000 RCF for 1 h at 4 °C. The clarified supernatant after the two ultracentrifugations was reserved as the cytosolic fraction “C”. The remaining “M” pellets were combined, resuspended in 250 µl of hypotonic buffer, and triturated using 10 passes through a 27 ½ G syringe. The resuspended “M” fraction was centrifuged for 45 min at 96,000 RCF at 4 °C. The supernatant from this centrifugation was combined with the cytoplasmic fraction “C”. The remaining “M” pellet was resuspended in 250 µl of hypotonic buffer, combined with the nuclear “N” fractions, and solubilized by adding NP-40 at a final concentration of 0.5% to create a mixed “M” + “N” fraction. The combined “M” + “N” fraction was centrifuged twice at 2,655 RCF to pellet nuclear membranes. The nuclear pellets were combined in 500 μl of hypotonic buffer and sonicated to solubilize for the final nuclear “N” fraction. The supernatant from this centrifugation was the final membrane “M” fraction. Samples were prepared with Laemmli sample buffer + 0.1 mM DTT and assessed via SDS-PAGE on 9% gels. Proteins were transferred to PVDF. Membranes were blocked for 1 h in 4% BSA and probed for rabbit anti-Na^+^/K^+^ ATPase (Cell Signaling Inc, 1:1,000, Cat #3010S, RRID: AB_2060983), mouse anti-P84 (GeneTex, 1:2000, Cat # GTX70220, RRID: AB_372637), mouse anti-GAPDH (GeneTex, 1:50,000, Cat # GTX627408, RRID: AB_11174761), and rabbit anti-GLUT1 (abcam, 1:200, Cat # ab15309, RRID: AB_301844). Blots were analyzed by densitometry in Image J v1.52i (https://imagej.nih.gov/ij/download.html).

### Western blotting

After pulse experiments, serum starvation, hormone treatments, or pituitary dissection, cells/tissues were lysed in RIPA/NP-40 buffer (Cell Signaling) supplemented with 1 mM phenylmethylsulfonyl fluoride, protease inhibitor cocktail, and phosphatase inhibitor cocktail (MedChemExpress). Samples were prepared in Laemmli buffer at 65 °C for 10 min to reducing smearing of glycosylated proteins and separated by SDS-PAGE on 10% gels and transferred onto 0.45 µm nitrocellulose membranes (BioRad). Membranes were blocked in Pierce StartingBlock (PBS) Blocking Buffer (Thermo Scientific) for 1 h at room temperature and incubated with primary Ab: rabbit anti-GLUT1 (abcam, 1:200, Cat # ab15309, RRID: AB_301844), rabbit GnRHR (Proteintech, 1:1,000, Cat # 19950-1-AP, RRID: AB_10644155), or mouse anti-β-Actin (Cell signaling, 1:1,000, Cat # 3700S, RRID: AB_2242334) overnight at 4 °C, then in 1:5,000 dilution of HRP-conjugated secondary antibody (Santa Cruz Biotechnology, goat anti-rabbit IgG-HRP: sc-2004, RRID: AB_631746 and goat anti-mouse IgG-HRP: sc-2005. RRID: AB_631736) at room temperature for 1 h. Blots were visualized by chemiluminescence and imaged using a Pxi system (Syngene). After imaging, blots were stripped in Restore Western Blot Stripping buffer (Thermo Scientific) for 15 min and reblotted with a subsequent primary antibody at 4 °C overnight. Quantitative densitometry was performed using Image J software v1.52i (https://imagej.nih.gov/ij/download.html). Data from at least 3 independent experiments were normalized to Actin and reported as fold change relative to the control.

### Quantitative reverse transcriptase PCR

Total mRNA from LβT2 cells, LβT2 transduced cell lines, and primary pituitary cells were isolated using TRIzol (Thermo Fisher Scientific) or RNeasy Plus Micro Kit (Qiagen). cDNA was synthesized using High Capacity cDNA Reverse Transcription Kit per the manufacturer’s instructions (Applied Biosystems). Quantitative reverse transcriptase PCR was carried out using the CFX Connect Real-Time PCR Detection System (Bio-Rad Laboratories) with KAPA SYBR Green PCR kit (KAPA Biosystems) supplemented with 200 nM of transcript-specific primers (Table 2) with cycling conditions recommended by the manufacturer. Reaction efficiency was determined for each primer set. A melt curve was performed after each PCR run to ensure that a single product was amplified. At least three independent determinations of mRNA content were made, and the relative transcript levels were determined with *Gapdh* mRNA as an endogenous control.

**Table 2:** Primer sequences used for quantitative reverse transcriptase PCR.

Gene	Sequence 5′ → 3′		Primer efficiency
	Forward	Reverse	
*Slc2a1*	ACTGGGCAAGTCCTTTGAG	CATGATGGAGTCTAAGCCAAAC	1.88
*Slc2a2*	GCCTAAAACCGAGGAACCGA	TACTCCTGGGTGTAGTCGCA	1.90
*Slc2a3*	GTGGAGCGGTGAAGATCAGA	CGAAGCCCAGCCTACCTATT	2.30
*Slc2a4*	GCCCGGACCCTATACCCTAT	GGGTTCCCCATCGTCAGAG	1.93
*Slc2a5*	GACTCTGCGTGTCTGCAGTT	GCCCACTTAGGACCTCCTTT	1.47
*Slc2a6*	GTGCTGGGCAATTTCAGCTT	TGAACACGGACCCAAACCAG	1.94
*Slc2a7*	CAAGCAACAGATGGGGTCCT	CCATGAATGTTCCGTGTCGC	> 2.5*
*Slc2a8*	TGTCGGGTGTGATCATGGTG	CTACGTGGGAGGAGTTGCTG	1.98
*Slc2a9*	AGATGCCCTGGCAAGTCC	AGGACCATTTCTTTGTCCTCCT	1.81
*Slc2a10*	GTGACAAGTGCTAGGACCCC	ACCAACACACGGATGAGTCC	2.11
*Slc2a12*	TGGCTTTTACCCACGGGATG	ATTAGGGAGGGGGTCTGGTC	2.28
*Egr1*	ATTTTTCCTGAGCCCCAAAGC	ATGGGAACCTGGAAACCACC	2.18
*Cfos*	GGCAAAGTAGAGCAGCTATCTCCT	TCAGCTCCCTCCTCCGATTC	2.16
*Lhb*	TGTCCTAGCATGGTCCGAGT	CCCCCACAGTCAGAGCTACT	2.04
*Txnip*	GGACTACTTGCGCTATGAAG	TTCACCCAGTAGTCTACGCA	1.98
*Gapdh*	TGCACCACCACCTGCTTAG	GGATGCAGGGATGATGTTC	2.02

*All primers were validated by PCR product analysis on an agarose gel and melt curve analysis. Low expression of several *Slc2a* members precluded accurate determination of reaction efficiency. An assumed efficiency of 100% was used for analysis for these *Slc2a* members*.*

### GnRH pulse stimulation and LH secretion assay

For perfusion experiments as previously described^25^, LβT2 cells were seeded on cytodex 3 microcarrier beads (GE Healthcare, Buckinghamshire, UK) at a density of 1.5 × 10^7^ cells/ml bead volume. After culture for 24 h, cells were pelleted by spinning at 550 RCF for 1 min, resuspended in 10 ml serum-free DMEM with antibiotics, repelleted, and placed in 10 ml serum-free DMEM for 16 h. Cells were then loaded into perifusion columns and equilibrated for 1 h in serum-free, phenol-red free DMEM supplemented with penicillin and streptomycin at a flow rate of 200 µl/min. Subsequently, cells were pulsed for 2 min with 40 nM GnRH for a final amplitude of 10 nM as determined by dilution of phenol red tracer dye at 58-min intervals for 4 h. Secreted LH concentrations in perifusate fractions collected every 5 min during the experiment were measured using the MILLIPLEX MAP Kit (Mouse Pituitary Magnetic Bead Panel, Cat. No. MPTMAG-49K, EMD Millipore Co., Charles, Missouri) following the manufacture’s instruction. Secreted LH was normalized to total protein post pulse. Area under the curve (AUC) and mean amplitude were calculated after subtracting the average baseline LH concentration per samples from each respective dataset. Each pulse experiment was performed a minimum of three independent times.

### LH surge model

To induce the LH surge^43,67,68^, female C57BL/6 mice underwent bilateral ovariectomy. LH surge was induced through one of two paradigms. For most animals, five days after surgery, animals were given a subcutaneous injection of 0.25 ug of *β*-estradiol benzoate (EB; Sigma-Aldrich) in 100 μl of sesame oil 4 h after lights on. On the following day, animals were given 1.5 μg of EB in sesame oil (100 μl) 4 h after lights on. The following day, animals were euthanized at lights off and pituitaries were snap frozen on dry ice and blood collected and processed to serum. Sera was processed using a Luminex assay and the Millipore MAGPIX as described, and an LH surge was considered a value greater than 1.5 ng/ml. The LH surge in three animals was induced by ovariectomy and E2 pellets as described^43^. There is no difference in the amplitude of the LH surge between these paradigms^43^. For this paradigm, LH in serum samples was measured via Luminex or via the University of Virginia Ligand Core, and an LH surge was accepted at a concentration of 0.6 ng/ml. Pituitaries where homogenized (PRO Scientific Bio-Gen PRO200 Homogenizer), then lysed in RIPA/NP-40 lysis buffer. Western blots were performed on pituitary lysates.

### Statistics

All experiments were repeated at least three times independently, and reported values are presented as the means ± standard error of the mean. JMP software v14.0 (SAS Institute, https://www.jmp.com/en_us/home.html) was used to perform all statistical analysis. Analysis was performed on raw, normalized values or data Box Cox transformed to correct for heteroscedasticity. Data were evaluated by ANOVA and appropriate post hoc testing unless otherwise indicated. A value of p < 0.05 as indicated was considered significant. Asterisks denote significance from the control. Data sharing a letter are not significantly different from each other, while data marked by letters exclusive to another data point are significantly different from each other.

## Supplementary information

Supplementary Information

## Data Availability

The authors confirm that the data supporting the findings of this study are available within the article and its supplementary materials and are available upon request.
