# Supplementary material for: GLUT1-mediated glycolysis supports GnRH-induced secretion of luteinizing hormone from female gonadotropes
Source: Sci Rep. 2020 Aug 3;10:13063. doi: 10.1038/s41598-020-69913-z (PMC7400764; doi:10.1038/s41598-020-69913-z)
Supplement: Supplementary file 1 — Supplementary Information [file 41598_2020_69913_MOESM1_ESM.pdf]

GLUT1-mediated Glycolysis Supports GnRH-induced Secretion of Luteinizing Hormone from Female Gonadotropes

Supplemental Figures

Dequina A. Nicholas[1], Vashti S. Knight[1], Karen J. Tonsfeldt[1], Tomohiro Terasaka[1], Olivia Molinar-Inglis[2], Shannon B. Z. Stephens[1], JoAnn Trejo[2], Alexander S. Kauffman[1], Pamela L. Mellon[1], Mark A. Lawson[1*]

[1]Department of Obstetrics, Gynecology and Reproductive Sciences, and Center for Reproductive Science and Medicine, University of California, San Diego, La Jolla, California

[2]Department of Pharmacology, University of California, San Diego, La Jolla, California

*Corresponding Author: mlawson@ucsd.edu

Nicholas, et. al. Figure S1

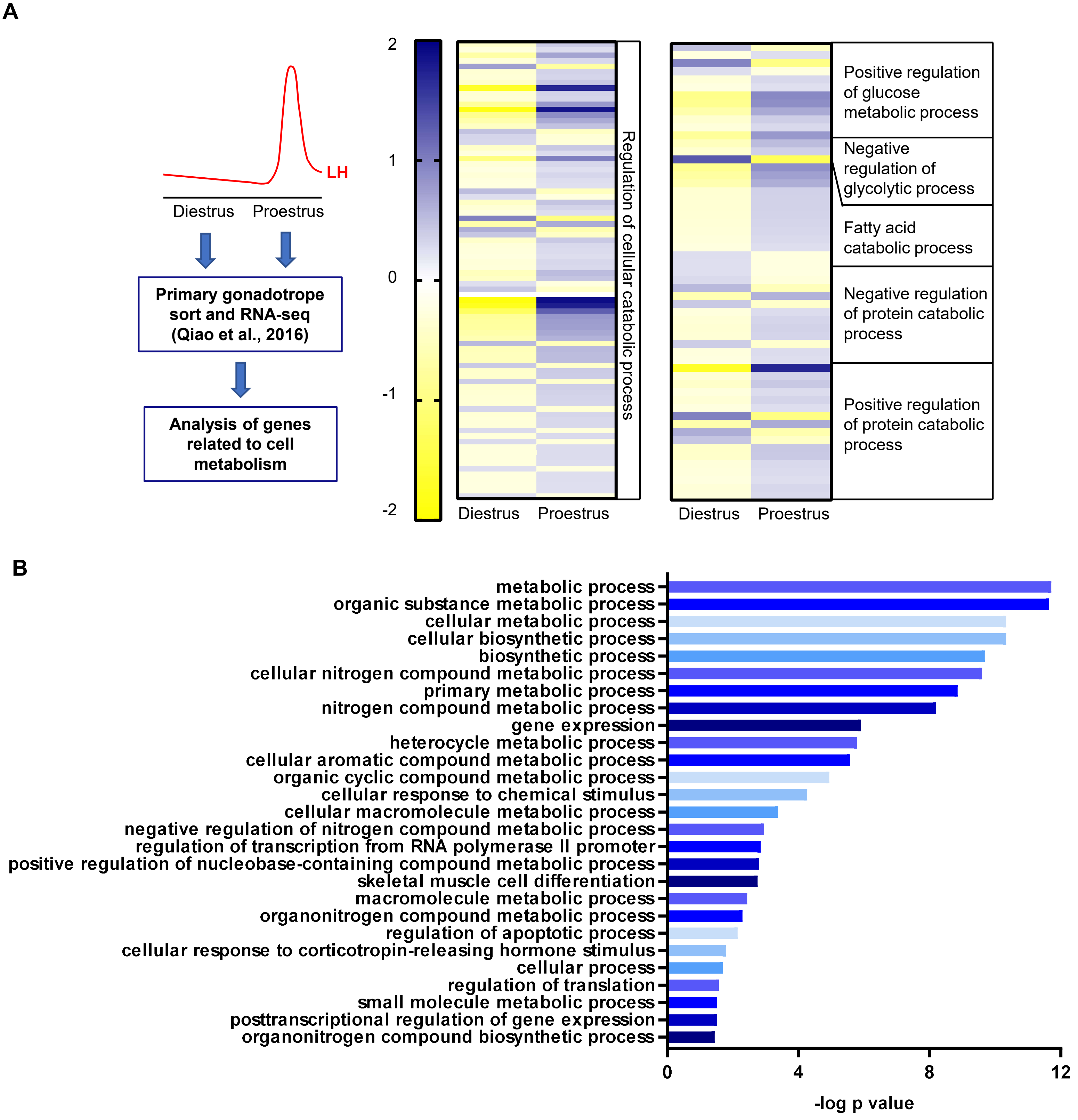

Figure S1. Related to Table 1 and Figure 1. Transcriptome analysis reveals increased metabolism is associated with GnRH stimulation of gonadotropes. (**A**) Heat maps of genes involved in cellular metabolism. Published RNA-seq data (Qiao et al., 2016) of sorted primary gonadotropes from female mice in diestrus and proestrus were independently analyzed. Data are expressed as mean FPKM, log2 transformed, and mean centered. Blue, relatively high expression; Yellow, relatively low expression. (**B**) Bar graph of Gene Ontology enrichment with GnRH treatment in LβT2 cells. Published Bulk RNA-seq dataset (GSE42120 - LβT2 cells treated +/- 45 min GnRH) was analyzed for functional enrichment of GO-terms with DAVID and summarized using REVIGO (Wang et al., 2013; Supek et al., 2011)(http://revigo.irb.hr/). The –log p value is indicated for the enriched GO-terms remaining after redundancy reduction.

Nicholas, et. al. Figure S2

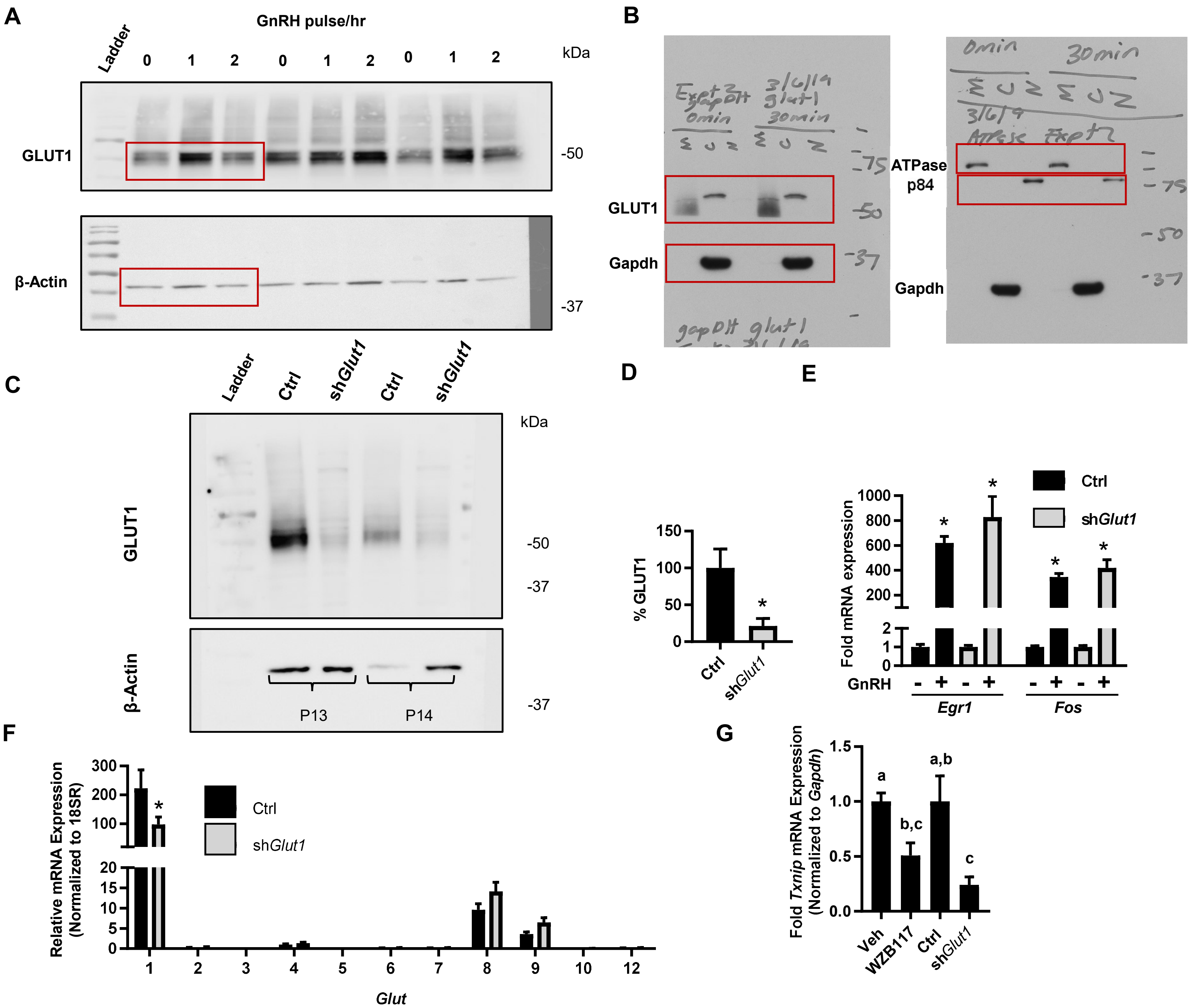

Figure S2. Related to Table 2, Figure 1, and Figure 4. Knock-down of GLUT1 does not impact GnRH signaling or induce GLUT expression compensation. (**A**) Protein expression of GLUT1 in response to increased frequency of GnRH pulsatility (N=3), full blot. Red boxes are cropped blots in Figure 1B. LβT2 cells were pulsed with a 10 nM amplitude of GnRH either once or twice per hour for four hours. Protein lysates were assessed by western blot. (**B**) Example uncropped blot from Figure 1D, red boxes indicate cropping in Figure 1D. LβT2 cells ± 30 min 10 nM GnRH were fractionated into cellular components and analyzed by western blot. Each experiment was run on one gel, the membrane cut, then probed simultaneously for all targets. (**C**) Western blot of GLUT1 knockdown in sh*Glut1* compared to Ctrl (non-targeting shRNA) transduced LβT2 cell lines from 2 separate passages run on one gel. (**D**) Quantification of GLUT1 Knock-down in sh*Glut1* transduced cell lines by western blot analysis (N=3). (**E**) Controls and sh*Glut1* cells were stimulated with GnRH for 15 min after serum starvation. Isolated mRNA was analyzed by qPCR for *Egr1* and *Fos*. (**F**) Relative expression of *Glut* family mRNA in control and sh*Glut1* LβT2 cell lines. Cells were cultured for 24 hrs and serum starved overnight before mRNA isolation and qPCR. Significance was determined by t Test on Box-Cox transformed values. (**G**) After pulse experiments (Figure 4) mRNA was extracted from LβT2 cell lines. Quantitative PCR of *Txnip* was performed. Significance was determined by Dunnet's test of Box Cox transformed values.

Nicholas, et. al. Figure S3

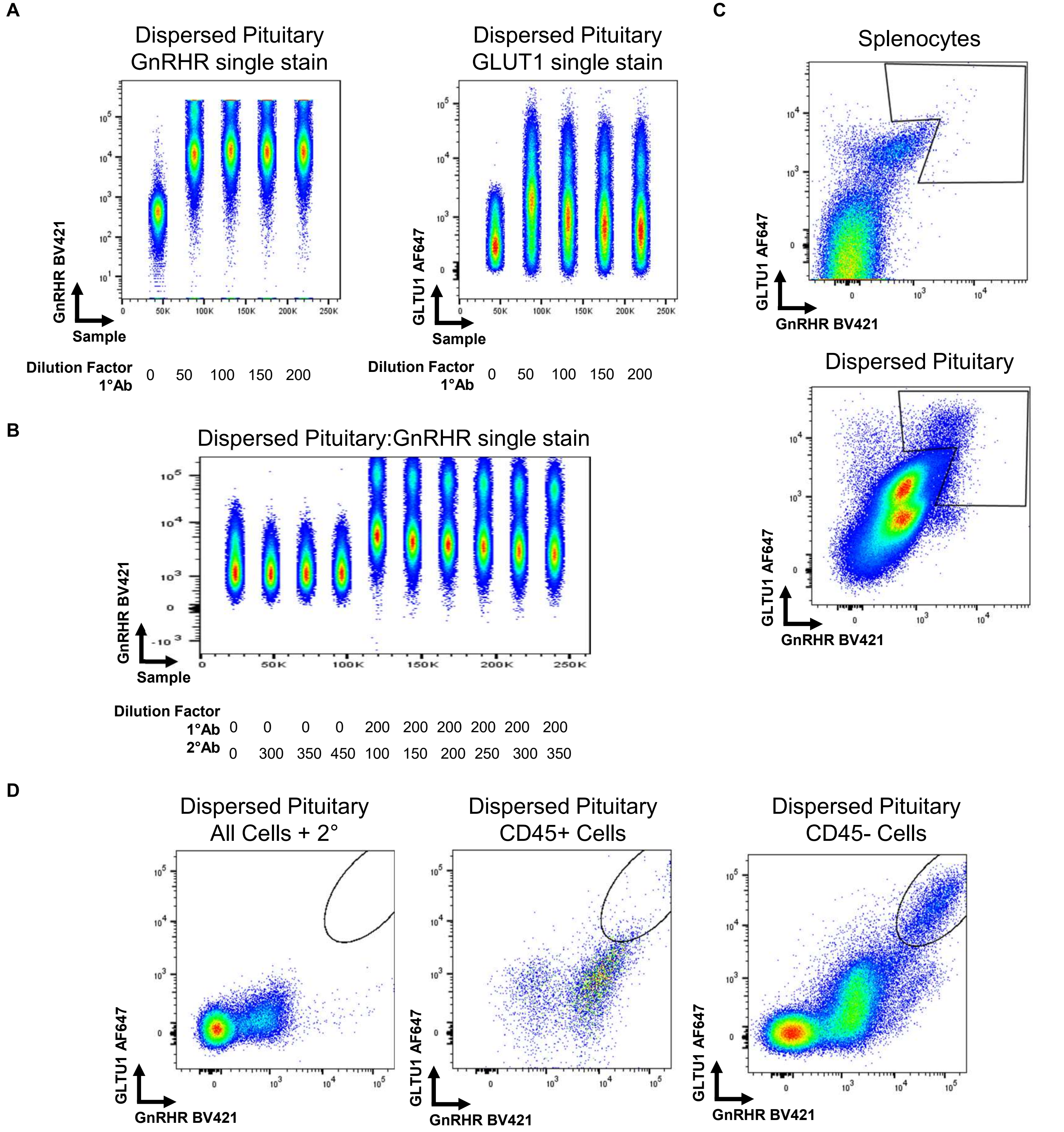

Figure S3. Related to Figure 5 and 7. Validation of GnRHR antibody and FAC sorting gating strategy for primary gonadotropes. (**A**) Concatenated fcs files of antibody titrations for GnRHR, Left (anti-rabbit BV421 used at 1:100 for all 1° GnRHR Ab titrations) and GLUT1, Right. (pooled 10 wk C57BL/6 male mice) (**B**) Concatenated fcs files of antibody titrations for GnRHR (Ab concentrations are indicated, pooled 10 wk C57BL/6 male mice) (**C**) Dissociated pituitary and splenocytes from 10 wk C57BL/6 female mice were stained with anti-GLUT1 and anti-GnRHR. Splenocytes (top) do not express GnRHR and are a biological control to account for cross reactivity of goat anti-rabbit BV421 2°Ab with GLUT1 AF647 Ab raised in rabbit. Gates set from the biological control (top) allow for gating on GnRHR+ cell in pituitary samples (bottom). (**D**) Internal biological control (CD45+ cells or leukocytes) are an endogenous sample gating control in addition to FMOs and unstained samples. GLUT1+ staining is determined by setting gates on the unstained control with 2° and viability dye (Left). GnRHR+ staining is determined by adjusting gates relative to CD45+ cells (Center) which are largely GnRHR negative. This gate is then applied to CD45- cells from the pituitary to sort gonadotropes (Right).

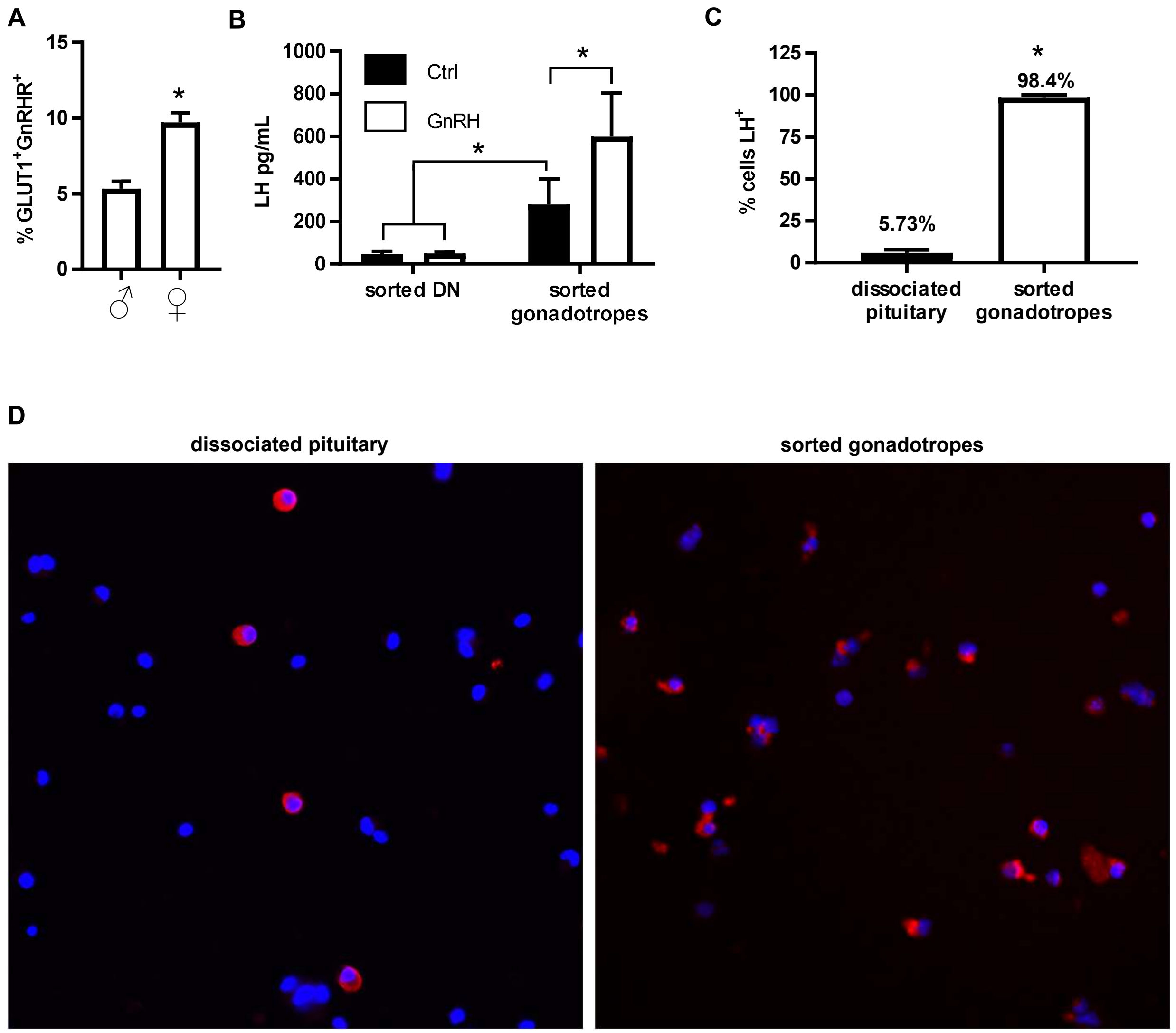

Figure S4. Related to Figure 5. FAC sorting dispersed pituitary by co-expression of GLUT1 and GnRHR yields pure and viable gonadotropes. (**A**) Percent of GLUT1^{+}GnRHR^{+} cells in male (N=6) and female (N=7) pituitary. (**B**) Sorted DN (GLUT1^{-}GnRHR^{-},double negative, N=9) and sorted gonadotropes (GLUT1^{+}GnRHR^{+}, N=5) were stimulated with 10nM GnRH for 30 min after overnight culture and a 1 hr serum starvation. After GnRH treatment, the conditioned media was analyzed by Luminex for LH. Significance was determined by Dunnet's Test comparing to ctrl sorted gonadotropes. (**C and D**) Dissociated pituitary and sorted gonadotropes were plated on lysine coated slides and stained with anti-LH and DAPI. Gonadotrope purity was calculated and plotted from images such as (D). 98.4% of the sorted gonadotropes were LH^{+} (2 experiments, data is mean %LH+ of 4 images [dissociated pituitary] and 11 images [sorted +/+ gonadotropes]). Significance was determined by t Test.

Nicholas, et. al. Figure S5

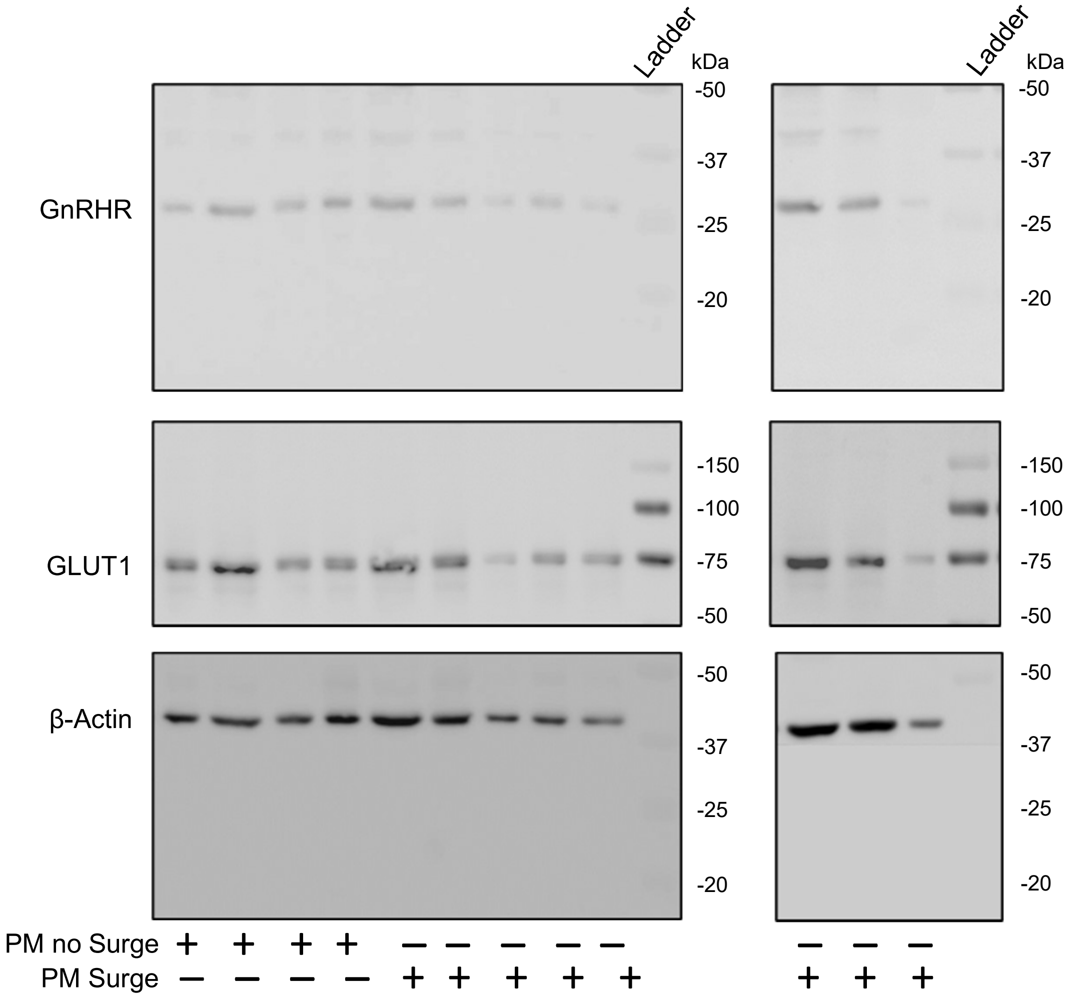

Figure S5. Related Figure 7. Western blot images of data presented in Figure 7. LH surge was induced in female mice by ovariectomy and estrogen treatment. Pituitary and blood (serum) was collected on the evening of the surge. Serum LH concentrations < 0.6 ng/mL is no Surge, N=4. Concentrations >1.5 ng/mL is Surge, N=8. Pituitaries were lysed and western blots for the indicated targets were performed. Samples were run on two gels as indicated by the cropping. After cutting the blots in half at 50kDA, each target was probed from the same membrane after stripping previous antibodies. A control sample was run on each blot to allow for comparison across blots.
